# New Experimental Conditions for Diels–Alder and Friedel-Crafts Alquilation Reactions with Thiophene: A New Selenocyanate with Potent Activity against Cancer

**DOI:** 10.3390/molecules27030982

**Published:** 2022-02-01

**Authors:** Gorka Calvo-Martín, Daniel Plano, Carmen Sanmartín

**Affiliations:** 1 Departamento de Tecnología y Química Farmacéuticas, Facultad de Farmacia y Nutrición, Universidad de Navarra, Irunlarrea 1, E-31008 Pamplona, Spain; gcalvo.3@alumni.unav.es; 2 Unidad de Onco-Hematología, Instituto de Investigación Sanitaria de Navarra (IdiSNA), Irunlarrea 3, E-31008 Pamplona, Spain

**Keywords:** thiophene, selenophene, Diels–Alder, cycloadition, Friedel–Crafts alquilation, selenium, cancer

## Abstract

The reactivity of thiophene in Diels-Alder reactions is investigated with different maleimide derivatives. In this paper, we have synthesized for the first time the Diels–Alder adducts of thiophene at room temperature and atmospheric pressure. Maleimido–thiophene adducts were promoted by AlCl_3_. The effects of solvent, time, temperature and the use of different Lewis acids were studied, showing dramatic effects for solvent and Lewis acid. Furthermore, the catalysis with AlCl_3_ is highly stereoselective, preferably providing the exo form of the adduct. Additionally, we also discovered the ability of AlCl_3_ to catalyze the arylation of maleimides to yield 3-aryl succinimides in a straightforward manner following a Friedel–Crafts-type addition. The inclusion of a selenocyanate group contributes to the cytotoxic activity of the adduct. This derivatization (from compound **7** to compound **15**) results in an average GI_50_ value of 1.98 µM in the DTP (NCI-60) cell panel, resulting in being especially active in renal cancer cells.

## 1. Introduction

The Diels–Alder (DA) reaction is a very useful tool in organic chemistry, commonly used since the discovery made by Otto Diels and Kurt Alder in 1928. The versatility and simplicity of this [4 + 2] cycloaddition is used in many synthetic steps of several drugs and natural products, such as cortisone, reserpine or cholesterol [1]. Thiophene is an heteroaromatic cycle, analog of furan, where oxygen is substituted by sulfur. The replacement of oxygen by sulfur has a significant impact on the reactivity of thiophene compared with furan [2]. Furan has a low aromatic character, hence being a good diene for the Diels-Adler reaction. On the contrary, thiophene is much more aromatic and, consequently, a poor diene. The reaction energy of thiophene is too high for a thermal cycloaddition but can be diminished by the introduction of substituents on the S atom, with the subsequent loss of aromaticity [3].

Thiophene derivatives, such as thiophene *S*-oxide, thiophene-*S,S*-dioxide or 2,5-dimethoxythiophene, can react as diene by thermal induction [4,5] or as dienophiles [6]. Currently, the cycloaddition of thiophene with maleic anhydride, maleimides, or other activated dienophiles can be promoted only at very high pressures above 10 Kbar [7,8]. The first successful attempt was in 1972, when the DA reaction of thiophene and dicyanoacetylene was conducted at 120 °C for 2 days with low yield (8%). When other less reactive dienophiles were used, such as maleic anhydride, high pressure was required [9]. The most successful attempt so far was conducted by Kotsuki et al. A pressure of 0.8 GPa and solvent free condition at 100 °C was needed for the formation of different DA adducts with various dienophiles and high yields of about 95%. A dramatic effect on solvent and pressure was found. Thiophene–maleimide Diels–Alder adducts were formed with poor stereospecificity [10]. On the other hand, thiophene and its analogs [11] show interesting properties in medicinal chemistry, especially in cancer. In addition, many DA adducts are known for their antitumoral effects [12,13]. The synthetic drawbacks for obtaining thiophene DA adducts have been limited to a few biological studies [14]. In this context, exploring effective new and accessible synthetic strategies for these adducts is justified.

The use of Lewis acid as a catalyst is a useful tool for the synthesis of DA adducts [15,16,17]. In this paper, we report unprecedented thiophene–maleimido adducts obtained at atmospheric pressure promoted by aluminum trichloride (AlCl_3_). These results provide an easy way to synthesize thiophene DA adducts avoiding the use of high-pressure conditions or expensive reagents. 

## 2. Results and Discussion

### 2.1. Synthesis Optimization

**Scheme 1:**
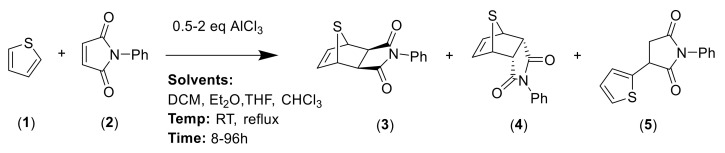
General scheme of synthesis for Diels–Alder adducts **3** and **4** and for byproduct **5**.

In order to optimize the synthetic procedure, several Lewis acids, solvents and conditions were explored. The DA reaction of thiophene 1 with *N*-phenylmaleimide 2 under different conditions yielded three compounds: the two isomers exo **3** and endo **4** and the formation of a byproduct **5** (Scheme 1). Initially, we studied the influence of the solvent comparing the effect of diethyl ether (Et_2_O), tetrahydrofuran (THF), chloroform (CHCl_3_) and methylene chloride (DCM). Experiments were carried out at both room temperature and reflux. Different ratios for the reagents and reaction times were also tested. Experimental conditions for the optimization of this reaction are summarized in Table 1.

The solvent has a dramatic effect on the reaction. The use of solvents, such as Et_2_O or THF, where the AlCl_3_ has better solubility, does not lead to any product, recovering only the *N*-phenylmaleimide. Chloroform, which should have similar behavior than methylene chloride (DCM), drives the reaction to higher amounts of the byproduct **5**. The equivalents of AlCl_3_ used were also pivotal. Only when one equivalent or less of AlCl_3_ were used, the main products were the DA adducts **3** and **4**. On the contrary, two equivalents of AlCl_3_ generated exclusively the byproduct **5** and no DA adduct was detected. In all the cases, the main DA adduct was the exo form **3**, presenting about a 9:1 rate of stereospecificity. 

Several Lewis acids were tested using the best reaction conditions found at 24 h, meaning: five equivalents of thiophene, DCM as solvent, room temperature and one equivalent of AlCl_3_ (entry 5 in Table 1). Results are summarized in Table 2. After using one equivalent of each Lewis acid, AlCl_3_ was the only one that afforded the reaction with acceptable yields. FeCl_3_, SnCl_4_ and BF_3_·H_2_0 gave no product, and Me_2_AlCl and TiCl_4_ only with very poor yields.

Additionally, different dienophiles were used to determine the applicability of these novel reaction conditions for the DA reaction of thiophene. The selected dienophiles contain at least one electron-withdrawing group, such as carbonyl or nitrile. One gram of each dienophile was placed in a round bottomed flask and dissolved in 60 mL of DCM. Then, five equivalents of thiophene were added, followed by one equivalent of AlCl_3_. The reaction was stirred at RT for 4 days. Afterwards, the reaction was quenched with water, extracted with DCM and dried over Na_2_SO_4_. The crude product was analyzed by TLC and q-^1^H-NMR using dimethyl sulfone as the internal standard. The results summarized in Table 3 show that only maleimide derivatives were able to undergo the DA reaction with thiophene. 

Maleimide derivatives are well-known dienophiles [18]. The two carbonyl groups on both sides conjugated with the double bond, and the cis conformation of the double bond activate this reagent towards the DA reaction. Due to the aromaticity and uniformity of the thiophene ring, this heteroaromatic compound is considered an unreactive diene. The methylation or oxidation of the S atom was proposed as the best way to disrupt this aromaticity, making the heterocycle reactive for the DA reaction [3,4,5].

In this particular case, we avoided the transformation of the thiophene ring. The proposed mechanism implies the coordination of AlCl_3_ with the oxygen atom of the carbonyl group of the maleimide, increasing the electrophilicity of the dienophile and preferably favoring the cycloaddition to the exo form [19,20].

#### Reaction Mechanism Discussion

**Scheme 2:**
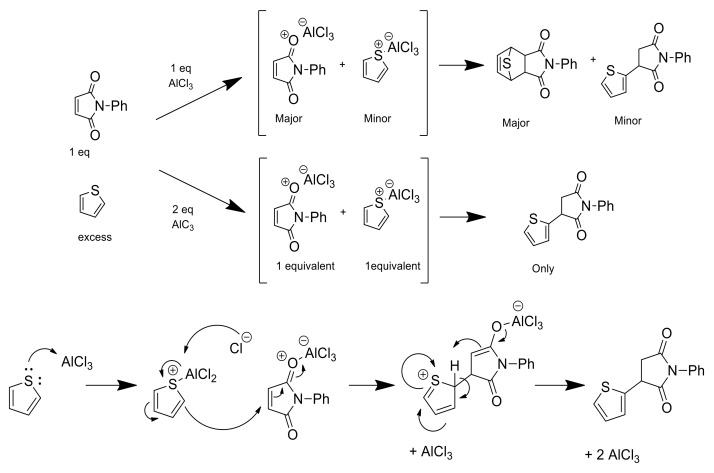
Proposed mechanism of reaction for the DA adducts or Friedel–Crafts-type products depending on the equivalents of AlCl_3_ used.

In order to further confirm the reaction mechanism proposed, different sequences of addition of the reagents were carried out. When 1 eq. of AlCl_3_ was added to a solution of *N*-phenylmaleimide in DCM, stirred for 30 min at room temperature and then filtered to remove unreacted AlCl_3_, the DA cycloaddition occurred after addition of thiophene to the filtrate. On the contrary, when thiophene was mixed first with AlCl_3_, after the subsequent filtration, no reaction was observed after adding the *N*-phenylmaleimide. Accordingly, AlCl_3_ shows preference to interact with the O-atom of the carbonyl group of the maleimide instead of the S-atom of the thiophene. When two equivalents of AlCl_3_ were added to a solution containing maleimide and thiophene, both were coordinated with AlCl_3_ carrying out the electrophilic attack of the thiophene cation to the maleimide yielding compound **5** as the main product (Scheme 2).

Encouraged by these results, we repeated the same optimal experimental protocol at 24 h (entry 5 in Table 1) but by adding 2 eq. of AlCl_3_ to a solution of *N*-phenylmaleimide and stirring the solution for 30 min prior to the addition of thiophene. In this case, the main product was the Friedel–Crafts-type alquilation (compound **5**), though the aggregate yield of the cycloadducts was 17.7%. Hence, the cycloaddition was produced because a large excess of maleimide-AlCl_3_ coordination complex was present prior to the addition of thiophene. These results suggest that the DA reaction is accomplished by coordination of AlCl_3_ to the maleimide.

We replicated the DA reaction using *N*-methylpyrrole instead of thiophene. In this case, no DA adduct was found. The reaction led almost immediately to the addition of the *N*-methylpyrrole to the position 3 of the maleimide, an analog of compound **5.** This Friedel–Crafts-type alquilation was previously reported using pyrroles and indoles [21]. This arylation of the maleimide can be a useful tool in the synthesis of some bioactive molecules, such as phensuximide or camphorataimide. Nowadays, selective arylation of maleimides with transition metals, such as Ru, Rh or Co, as catalysts, have also been reported [22,23,24].

**Scheme 3:**
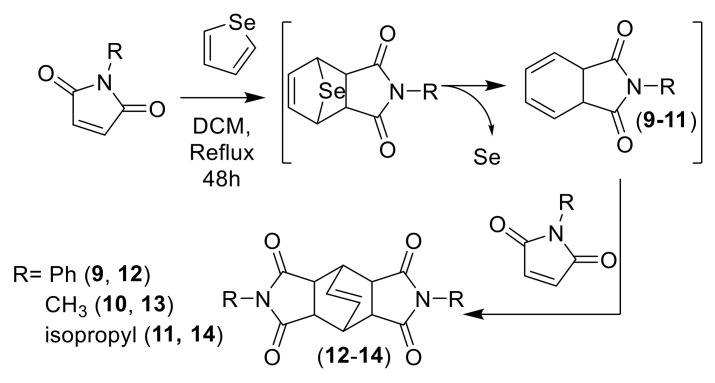
DA cycloaddition of selenophene with subsequent extrusion of selenium and followed by a second DA with maleimide to form “butterfly-like” diazatetradecenes (**12**–**14**).

Similar experiments were carried out employing selenophene as diene. In this case, an increment of temperature was necessary and the reaction mixture was refluxed for 48 h in DCM. Afterwards, the reaction mixture was quenched with water, extracted and the crude sonicated with Et_2_O. A precipitate was isolated as the main product of the reaction: a “butterfly-like” diazatetradecenes **12**–**14** as a result of a double DA cycloadduct formed through the intermediate dienes **9**–**11** (Scheme 3) by chelotropic extrusion of selenium, in the same way as it occurs with sulfur due to the instability of the selenium bridge [8,25].

### 2.2. Biological Evaluation

#### 2.2.1. Cytotoxicity Activity

**Scheme 4:**
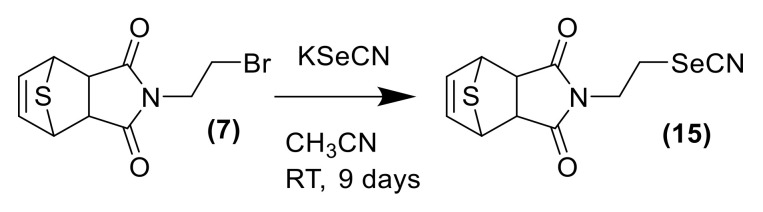
General scheme of synthesis for the selenocyanate derivative of thiophene DA adduct **15**.

**Figure 1:**
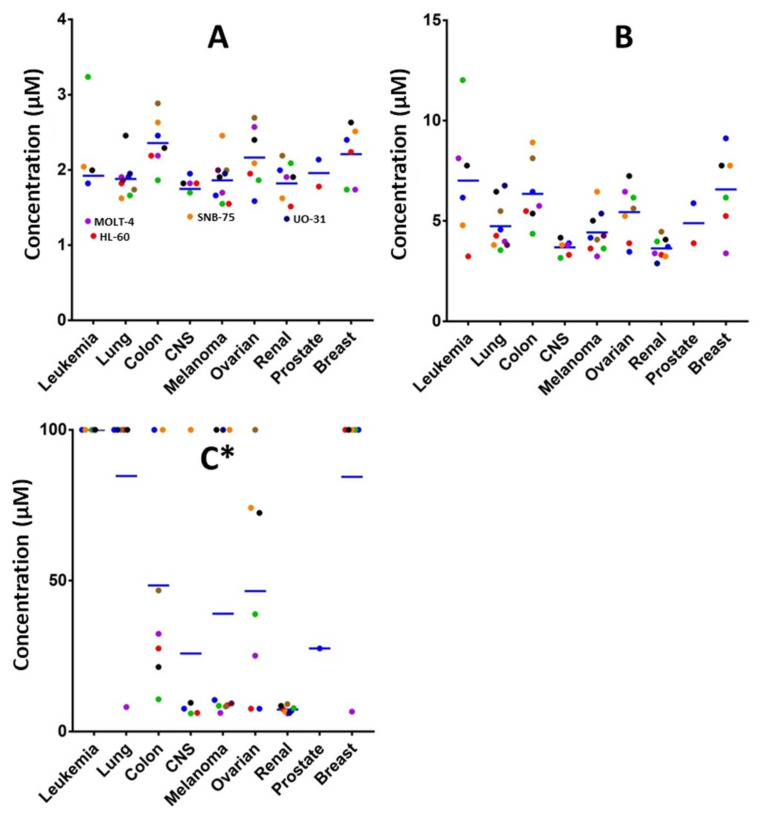
Representation of GI_50_ (**A**), TGI (**B**) and LC_50_ (**C**) [29] values of adduct **15** in the 60 cancer cell lines tested grouped by cancer type. * Values over 100 μM are represented as 100 µM. The LC_50_ values of some cell lines were not determined. Blue lines represent mean value for each cancer type.

In order to explore the potential biological efficacy of the adducts and considering the importance of selenocyanate framework in cancer research, the cycloadduct **7** was reacted with KSeCN to form cycloadduct **15** as shown in Scheme 4. The use of selenocyanate as cytotoxic scaffold for the development of new cytotoxic agents is well-known. The inclusion of the selenocyanate fragment in some drugs as the histone deacetylase Vorinostat [26,27] or the anti-inflammatory acetylsalicylic acid [28] afforded an exponential increase on the cytotoxic activity of the parent drugs. The novel cycloadduct was evaluated in a panel of 60 cancer cell lines by the DTP NCI-60 program with outstanding results (see Supplementary Materials). Derivative **15** shows an average GI_50_ value considering the 60 cell lines tested of 1.98 μM. This compound also shows impressive results in renal cancer cell lines, achieving LC_50_ values lower than 10 µM in all the renal cell lines tested, as shown in Figure 1C. Full cytotoxic information can be found in the supporting materials. 

#### 2.2.2. Compound **15** Induces an Arrest on the G2/M Phase of the Cell Cycle

**Figure 2:**
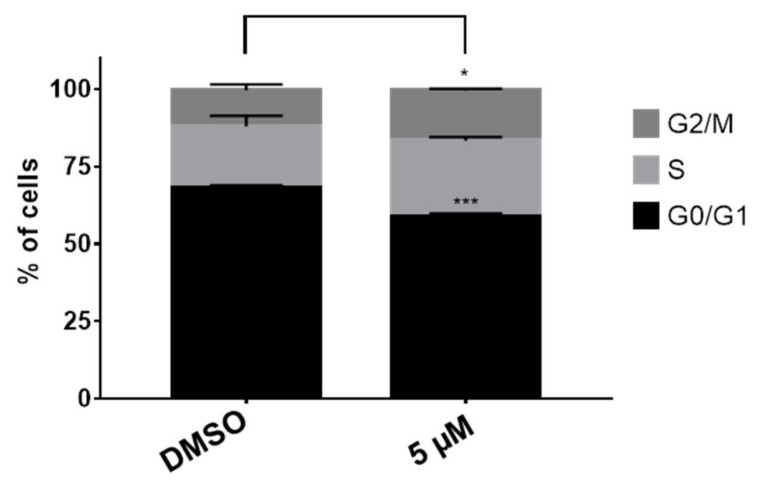
Cell cycle analysis of **15** in HT-29 cells. Percent of cells in different cell cycle stages treated with **15** at 5 µM and the vehicle (DMSO). Results are expressed as a mean ± SD of three independent experiments, * *p* ≤ 0.05 and *** *p* ≤ 0.001.

The modulation of the cell cycle is a common characteristic displayed by several cytotoxic agents. Usually, the cell cycle is arrested at G1/S and G2/M boundaries. With the aim to disclose the possible effect of compound **15** on the cell cycle, HT-29 colon cancer cells were treated with derivative **15** at 5 µM and compared with non-treated cell after 48 h. The cell cycle progression was monitored by flow cytometry employing propidium iodide as DNA dye. The results depicted in Figure 2 show a decrease of the G0/G1 phase and a significant increase of the G2/M phase after 48 h of treatment. These results suggest an arrest of the cell cycle at the G2/M boundary. This experiment was repeated at a higher dose (10 µM). Nonetheless, at this concentration, the number of dead cells was too high and the results were discarded. 

## 3. Materials and Methods

Commercial reagents were purchased from Merck (Darmstadt, Germany), Acros Organics (Geel, Belgium) and abcr-gute chemie (Karlsruhe, Germany) and used without further purification. ^1^H, ^13^C-NMR, and ^77^Se spectra were recorded on a Bruker NEO AVANCE 400 instrument (Karlsruhe, Germany) using CDCl_3_ as a solvent. Chemical shifts δ are expressed in parts per million (ppm) and internally referenced to tetramethylsilane (TMS). The coupling constant *J* is reported in Hz. The following abbreviations are used: s = singlet, d = doublet, t = triplet, q = quartet, and m = multiplet. High-resolution mass spectra (HRMS) were obtained using an Agilent 6220 Accurate-Mass Time-of-Flight mass spectrometer (Santa Clara, CA, USA). The ionization method chosen was electrospray ionization (ESI) operated in positive ion mode. All the reaction procedures were monitored by TLC using 40 precoated sheets of silica gel G/UV-254 of 0.25 mm thickness, Merck 60 F254 (BioLong, Yixing, China). TLC plates were visualized by exposure to ultraviolet light and/or by exposure to iodine vapors, and the product was obtained by chromatography performed on silica gel (200–300 mesh).

### 3.1. Synthesis

#### 3.1.1. Synthesis of 2-Bromoethyl Maleimide

4.10 g (20 mmol) of 2-bromoethylamine hydrobromide and maleic anhydride were placed in a round-bottom flask and stirred in 30 mL of DCM under N_2_ atmosphere. After 30 min, 2.8 mL (20 mmol) of triethylamine was added dropwise. After 24 h, the solution was washed with 3 × 20 mL of HCl 1M and 3 × 20 mL of water, dried over Na_2_SO_4_ and the solvent evaporated under reduced pressure. The obtained 4-((2-bromoethyl)amino)-4-oxobut-2-enoic acid was isolated as white crystals. 3.33 g (15 mmol) of the resulting 4-((2-bromoethyl)amino)-4-oxobut-2-enoic acid and 1.23g (15 mmol) of sodium acetate were placed in a round-bottomed flask and 15 mL of acetate anhydride added. The mixture was stirred at 100 °C for 2 h. The solution was neutralized with NaHCO_3_ and extracted with 3 × 20 mL of DCM. Organic layers were dried over Na_2_SO_4_ and the solvent was evaporated using a rotary evaporator. The resulting brown syrup was purified by silica column chromatography with a mixture of hexane/ethyl acetate in a 3:1 ratio as eluent. The product was obtained as white crystals.

#### 3.1.2. Synthesis of *N*-3-Acetoxypropyl Maleimide

The synthetic procedure is similar to the synthesis of 2-bromoethyl maleimide, employing 4.36 g (20 mmol) of 3-bromoproylamine hydrobromide instead of 2-bromoethylamine hydrobromide. The resulting brown syrup was purified by silica column chromatography with a mixture of hexane /ethyl acetate in a 3:1 ratio as eluent. The first fraction was characterized as *N*-2-bromoethyl maleimide, the second fraction being the substitution product of the acetate in the place of the bromide. Longer reaction time or higher temperatures will lead the reaction to the second byproduct. Both products were obtained as white crystals.

#### 3.1.3. Diels–Alder Reaction of Thiophene with *N*-Phenylmaleimides Promoted by AlCl_3_

768 mg (5.8 mmol) of pulverized AlCl_3_ were added to a solution of 1 g (5.8 mmol) of *N*-phenylmaleimide and 2.30 mL of thiophene (5 equivalents) in 60 mL of dry dichloromethane. The mixture was stirred at RT for 4 days. Then, the reaction was quenched with water, extracted with methylene chloride and the organic layers washed with brine and dried over Na_2_SO_4_. The solvent was evaporated, and the adduct **3** was purified by column chromatography using a gradient of hexane/ethyl acetate ranging for 3:1 to 1:1. The product was obtained as white crystals.

#### 3.1.4. Synthesis of Friedel–Crafts-Type Alquilation Compounds

Friedel–Crafts-type alquilation compounds were synthesized following the same experimental procedure as the Diels–Alder adducts, employing two equivalents of AlCl_3_ instead of one. *N*-methylpyrrole was employed instead of thiophene for the synthesis of 3-(1-methyl-1*H*-pyrrol-2-yl)-1-phenylpyrrolidine-2,5-dione. After 1 h, the reaction was completed and the products were purified by column chromatography using a mixture of hexane/ethyl acetate 3:1 as eluent. Resulting products were obtained as dark brown oil.

#### 3.1.5. Synthesis of “Butterfly-Like” Diazatetradecenes

6 mmol of the corresponding maleimide (*N*-phenyl, *N*-methyl or *N*-isopropyl maleimide) and 800 mg (6 mmol) of AlCl_3_ were placed in a round-bottom flask and dissolved in 25 mL of DCM. Then, 1.5 equivalents (9 mmol) of selenophene were added to the mixture and refluxed for 24 h. Then, the crude was filtered, extracted with water/DCM, dried over Na_2_SO_4_ and the solvent was evaporated in a rotary evaporator. The resulting oil was sonicated with diethyl ether and the obtained precipitate was filtered and washed with diethyl ether. Products were obtained as brownish powder.

#### 3.1.6. Synthesis of the Selenocyanate Derivative of Thiophene DA Adduct 15

250 mg (0.87 mmol) of cycloadduct **7** and 187 mg (1.3 mmol) of potassium selenocyanate were dissolved in 15 mL of acetonitrile and stirred for 9 days at room temperature. The solvent was removed in a rotary evaporator and the resulting solid was dissolved in DCM and washed 3 times with water and brine. The organic layer was dried with Na_2_SO_4_, the solvent was removed, and the resulting product purified by column chromatography hexane/ethyl acetate 1:1 to yield the pure product as a yellow solid.

### 3.2. Characterization Data

*(3aR,4R,7S,7aS)-2-phenyl-3a,4,7,7a-tetrahydro-1H-4,7-epithioisoindole-1,3(2H)-dione.* (**3**). White powder. Yield 33%. mp: 185–188 °C. IR (KBr) ῡ cm^−1^: 3463, 3063, 2976, 1775, 1709, 1593, 1493, 1392, 1204. ^1^H-NMR (400 MHz, CDCl_3_) δ: 7.44–7.36 (m, 2H, Ph), 7.36–7.30 (m, 1H, Ph), 7.22–7.17 (m, 2H, Ph), 6.57 (t, *J* = 2.1 Hz, 2H, H5 + H6), 4.50 (t, *J* = 2.1 Hz, 2H, H4 + H7), 3.24 (s, 2H, H3a + H7a). ^13^C-NMR (101 MHz, CDCl_3_) δ: 175.3 (C1 + C3), 140.1 (C5 + C6), 132.0 (Ph), 129.3 (Ph), 129.0 (Ph), 126.7 (Ph), 54.0 (C4 + C7), 50.3 (C3a + C7a). HRMS calcd. for C_14_H_11_NO_2_S [M + Na]: 280.0403, found: 280.0393 [M + Na].

*(3aR,4R,7S,7aS)-2-methyl-3a,4,7,7a-tetrahydro-1H-4,7-epithioisoindole-1,3(2H)-dione.* (**6**). White powder. Yield 25%. mp:144–146 °C. IR (KBr) ῡ cm^−1^: 3443, 3070, 2965, 1770, 1694, 1429, 1379, 1285. ^1^H-NMR (400 MHz, CDCl_3_) δ: 6.57 (t, *J* = 2.1 Hz, 2H, H5 + H6), 4.44 (t, *J* = 2.1 Hz, 2H, H4 + H7), 3.14 (s, 2H, H3a + H7a), 2.98 (s, 3H, CH_3_). ^13^C-NMR (101 MHz, CDCl_3_) δ: 176.3 (C1 + C3), 139.8 (C5 + C6), 53.4 (C4 + C7), 50.4 (C3a + C7a), 25.0 (CH_3_). HRMS calcd. for C_9_H_9_NO_2_S [M+]: 195.0354, found: 195.1317 [M+].

*(3aR,4R,7S,7aS)-2-(2-bromoethyl)-3a,4,7,7a-tetrahydro-1H-4,7-epithioisoindole-1,3(2H)-dione.* (**7**). Yellow powder. Yield 36%. mp: 85–87 °C. IR (KBr) ῡ cm^−1^: 3449, 3066, 2958, 1771, 1700, 1440, 1400, 1340. ^1^H-NMR (400 MHz, CDCl_3_) δ: 6.58 (t, *J* = 2.1 Hz, 2H, H5 + H6), 4.46 (t, *J* = 2.1 Hz, 2H, H4 + H7), 3.90 (t, *J* = 7.1 Hz, 2H, H1´), 3.45 (t, *J* = 7.1 Hz, 2H, H2´), 3.18 (s, 2H, H3a + H7a). ^13^C-NMR (101 MHz, CDCl3) δ: 175.6 (C1 + C3), 139.7 (C5 + C6), 53.4 (C4 + C7), 50.3 (C3a + C7a), 40.3 (C1´), 26.7 (C2´). HRMS calcd. For C_10_H_10_BrNO_2_S [M + Na]: 311.1507, found: 311.1989 [M + Na].

*3-((3aR,4R,7S,7aS)-1,3-dioxo-1,3,3a,4,7,7a-hexahydro-2H-4,7-epithioisoindol-2-yl)propyl acetate.* (**8**). Yellow oil. Yield 7.1%, IR (KBr) ῡ cm^−1^: 3455, 3072, 2957, 1772, 1735, 1701, 1441, 1401, 1368, 1231. ^1^H-NMR (400 MHz, CDCl_3_) δ: 6.57 (t, *J* = 2.1 Hz, 2H, H5 + H6), 4.44 (t, *J* = 2.1 Hz, 2H, H4 + H7), 4.05 (t, *J* = 6.3 Hz, 2H, H3´), 3.58 (t, *J* = 7.0 Hz, 2H, H1´), 3.13 (s, 2H, H3a + H7a), 2.05 (s, 3H, CH_3_), 1.95–1.87 (m, 2H, H2´). ^13^C-NMR (101 MHz, CDCl_3_) δ 176.1 (C1 + C3), 171.1 (C=O), 139.9 (C5 + C6), 61.8 (C3´), 53.4 (C4 + C7), 50.2 (C3a + C7a), 36.0 (C1´), 26.7 (C2´), 21.0 (CH_3_). HRMS calcd. For C_13_H_15_NO_4_S [M + Na]: 304.0614, found: 304.0798 [M + Na].

*N-2-Bromoethyl maleimide*. White powder. Yield 60%. mp: 60–62 °C. IR (KBr) ῡ cm^−1^: 3441, 3163, 3093, 2969, 2931, 1706, 1449, 1413. ^1^H-NMR (400 MHz, CDCl_3_) δ: 6.73: (s, 2H, H3 + H4), 3.93 (t, *J* = 6.6 Hz, 2H, H1´), 3.51 (t, *J* = 6.6 Hz, 2H, H2´). ^13^C-NMR (101 MHz, CDCl_3_) δ: 170.1 (C2 + C5), 134.2 (C3 + C4), 39.1 (C1´), 28.2 (C2´). HRMS calcd. For C_6_H_6_NO_2_Br [M + H]: 203.9582, found: 203.9567 [M + H].

*N-3-acetoxypropyl maleimide*. White powder. Yield 36 %. mp: 49–50 °C. IR (KBr) ῡ cm^−1^: 3451, 3175, 3109, 2960, 1729, 1704, 1461, 1413, 1383, 1236. ^1^H-NMR (400 MHz, CDCl_3_) δ: 6.69 (s, 2H, H3 + H4), 4.03 (t, *J* = 6.2 Hz, 2H, H3´), 3.61 (t, *J* = 6.9 Hz, 2H, H1´), 2.02 (s, 3H, CH_3_), 1.96–1.88 (m, 2H, H2´). ^13^C-NMR (101 MHz, CDCl_3_) δ: 171.0 (C=O), 170.7 (C2 + C5), 134.3 (C3 + C4), 61.7 (C3´), 35.0 (C1´), 27.5 (C2´), 20.9 (CH_3_). HRMS calcd. For C_9_H_11_NO_4_ [M + Na]: 220.0580, found: 220.0590 [M + Na].

*1-Phenyl-3-(thiophen-2-yl)pyrrolidine-2,5-dione*. (**5**).Brown oil. Yield 85%. IR (KBr) ῡ cm^−1^: 3478, 3101, 3068, 2925, 1780, 1714, 1567, 1497, 1382. ^1^H-NMR (400 MHz, CDCl_3_) δ 7.52–7.46 (m, 2H, Ph), 7.44–7.38 (m, 1H, Ph), 7.34–7.29 (m, 3H, Ph+H5-tiophene), 7.10 (dt, *J* = 3.5, 1.1 Hz, 1H, H3-tiophene), 7.04 (dd, *J* = 5.1, 3.6 Hz, 1H, H4-tiophene), 4.47 (ddd, *J* = 9.6, 5.2, 0.9 Hz, 1H, H3pyrrolidine), 3.45 (dd, *J* = 18.4, 9.6 Hz, 1H, H4-pyrrolidine), 3.14 (dd, *J* = 18.4, 5.2 Hz, 1H, H4-pyrrolidine). ^13^C-NMR (101 MHz, CDCl_3_) δ: 175.4 (C=O), 174.4 (C=O), 138.4 (C2-tiophene), 131.9 (Ph), 129.4 (Ph), 128.9 (Ph), 127.4 (thiophene), 126.6 (Ph), 125.8 (thiophene), 125.7 (thiophene), 41.4 (C3-pyrrolidine), 37.4 (C4-pyrrolidine). HRMS calcd. For C_14_H_11_NO_2_S [M + Na]: 280.0403, found: 280.0408 [M + Na].

*1-Methyl-3-(thiophen-2-yl)pyrrolidine-2,5-dione.* White powder. Yield 13%. mp: 82–84 °C. IR (KBr) ῡ cm^−1^: 3096, 2920, 1694, 1437, 1384, 1283, 1120. ^1^H-NMR (400 MHz, CDCl_3_) δ: 7.19 (dd, *J* = 5.1, 1.3 Hz, 1H, H5-thiophene), 6.93–6.91 (m, 2H, H3-thiophene + H4-tiophene), 4.21 (dd, *J* = 9.4, 5.2 Hz, 1H, H3-pyrrolidine), 3.19 (dd, *J* = 18.3, 9.4 Hz, 1H, H4-pyrrolidine), 2.97 (s, 3H, CH_3_), 2.87 (dd, *J* = 18.2, 5.1 Hz, 1H, H4-pyrrolidine). ^13^C-NMR (101 MHz, CDCl_3_) δ: 176.5 (C2-pyrrolidine), 175.4 (C5-pyrrolidine), 138.4 (C1-thiophene), 127.2 (C4-thiophene), 125.6 (C5-thiophene), 125.4 (C3-thiophene), 41.2 (C3-pyrrolidine), 37.2 (C4-pyrrolidine), 25.3 (CH_3_). HRMS calcd. For C_9_H_9_NO_2_S [M^+^ + Na]: 218.0246, found 218.0270 [M^+^ + Na].

*3-(1-Methyl-1H-pyrrol-2-yl)-1-phenylpyrrolidine-2,5-dione.* Black oil. IR (KBr) ῡ cm^−1^: 3473, 3102, 3064, 2943, 1711, 1595, 1496, 1384, 1189. ^1^H-NMR (400 MHz, CDCl_3_) δ: 7.50–7.44 (m, 2H, Ph), 7.43–7.37 (m, 1H, Ph), 7.31–7.26 (m, 2H, Ph), 6.67 (dd, *J* = 2.4, 2.0 Hz, 1H, H4-pyrrole), 6.15–6.11 (t, *J* =2.8 Hz, 1H, H3-pyrrole), 6.08 (dd, *J* = 3.5, 1.5 Hz, 1H, H2-pyrrole), 4.27 (dd, *J* = 9.5, 4.9 Hz, 1H, H3-pyrrolidine), 3.76 (s, 3H, N-CH_3_), 3.32 (dd, *J* = 18.4, 9.5 Hz, 1H, H4-pyrrolidine), 3.09 (dd, *J* = 18.4, 4.9 Hz, 1H, H4-pyrrolidine). ^13^C-NMR (101 MHz, CDCl_3_) δ: 175.5 (C2-pyrrolidine), 174.9 (C5-pyrrolidine), 131.9 (Ph), 129.3 (Ph), 128.8 (Ph), 127.1 (C1-pyrrole), 126.6 (Ph), 124.1 (C4-pyrrole), 107.4 (C3-pyrrole), 106.2 (C2-pyrrole), 38.3 (C3-pyrrolidine), 35.6 (C2-pyrrolidine), 34.5 (N-CH_3_). HRMS calcd. For C_15_H_14_N_2_O_2_ [M^+^ + Na]: 277.0947, found 277.0963 [M^+^ + Na].

*2,6-Diphenyl-3a,4,4a,7a,8,8a-hexahydro-4,8-ethenopyrrolo[3,4-f]isoindole-1,3,5,7(2H,6H)-tetraone*. (**12**). Brown powder. Yield 9%. mp: 155 °C. IR (KBr) ῡ cm^−1^: 3470, 1710, 1498, 1380, 1190. ^1^H-NMR (400 MHz, CDCl_3_) δ 7.42 (m, 6H, H7 + H8), 7.18 (d, *J* = 7.4 Hz, 4H, H6), 6.36 (t, *J* = 3.6 Hz, 2H, H2), 3.97 (s, 2H, H1), 3.20 (s, 4H, H3). ^13^C-NMR (101 MHz, CDCl_3_) δ: 175.8 (C4), 131.6 (C5), 131.5 (C2), 129.4 (C7), 129.1 (C8), 126.4 (C6), 43.1 (C3), 34.1 (C1). HRMS calcd. For C_24_H_18_N_2_O_4_ [M+]: 399.1345, found 399.1773 [M+].

*2,6-Dimethyl-3a,4,4a,7a,8,8a-hexahydro-4,8-ethenopyrrolo[3,4-f]isoindole-1,3,5,7(2H,6H)-tetraone*. (**13**). Brown powder. Yield 11%. mp: 197–200 °C. IR (KBr) ῡ cm^−1^: 3432, 2955, 1770, 1695, 1434, 1381, 1318, 1282. ^1^H-NMR (400 MHz, CDCl_3_) δ: 6.09 (t, *J* = 3.1 Hz, 2H, H2), 3.76 (s, 2H, H1), 2.98 (s, 4H, H3), 2.89 (s, 6H, CH_3_). ^13^C-NMR (101 MHz, CDCl_3_) δ: 176.8 (C4), 131.1 (C2), 43.1 (C3), 33.5 (C1), 25.0 (C5). HRMS calcd. For C_14_H_14_N_2_O_4_ [M + Na]: 297.0846, found: 297.0804 [M + Na].

*2,6-Diisopropyl-3a,4,4a,7a,8,8a-hexahydro-4,8-ethenopyrrolo[3,4-f]isoindole-1,3,5,7(2H,6H)-tetraone.* (**14**)**.** Brown powder. Yield 12%. mp: 210–215 °C. IR (KBr) ῡ cm^−1^: 3446, 2973, 1768, 1698, 1456, 1365, 1226. ^1^H-NMR (400 MHz, CDCl_3_) δ 6.85 (t, *J* = 3.1 Hz, 2H, H2), 4.25 (quin, *J* = 6.9 Hz, 2H, H5), 3.72 (s, 2H, H1), 2.88 (s, 4H, H3), 1.28 (d, *J* = 6.9 Hz, 12H, H6). ^13^C-NMR (101 MHz, CDCl_3_) δ: 176.9 (C4), 130.8 (C2), 44.1 (C5), 42.7 (C3), 33.8 (C1), 19.2 (C6) HRMS calcd. For C_18_H_22_N_2_O_4_ [M^+^ + H]: 331.1613, found 331.1936 [M^+^ + H].

*(3aR,4R,7S,7aS)-2-(2-selenocyanatoethyl)-3a,4,7,7a-tetrahydro-1H-4,7-epithioisoindole-1,3(2H)-dione* (**15**). Yellow solid. Yield 51%. mp: 110–112 °C. IR (KBr) ῡ cm^−1^:3451, 3018, 2947, 2146, 1767, 1702, 1433, 1396. ^1^H-NMR (400 MHz, CDCl_3_) δ: 6.59 (t, *J* = 2.2 Hz, 2H, H5 + H6), 4.47 (t, *J* = 2.1 Hz, 2H, H4 + H7), 4.00 (t, *J* = 6.6 Hz, 2H, H1´), 3.29 (t, *J* = 6.6 Hz, 2H, H2´), 3.28 (s, 2H, H3a + H7a). ^13^C-NMR (101 MHz, CDCl_3_) δ: 175.8 (C1 + C3), 139.8 (C5 + C6), 101.0 (SeCN), 53.5 (C4 + C7), 50.3 (C3a + C7a), 39.2 (C1´), 25.4 (C2´). ^77^Se NMR (76 MHz, CDCl_3_) δ: 204.7 (SeCN). HRMS calcd. For C_11_H_10_N_2_O_2_SSe [M + Na]: 336.9520, found: 336.9540 [M + Na].

### 3.3. Biological Evaluation

#### 3.3.1. NCI-60 Human Tumor Cell Line 5-Dose-Response

Compounds were evaluated in the NCI’s human tumor 60-cell line screening. Data analysis was performed as described [29].

#### 3.3.2. Cell Cycle Assay

The colorectal cancer cell line HT-29 was purchased from ATTC (Lublin, Poland). A total of 6 × 10^5^ cells/well were seeded in a 6-well plate for 24 h and then treated with 5 and 10 μM of **15**, DMSO (control) or 10 μM camptothecin (positive control). Propidium iodide (PI) was employed as DNA stain dye to determine cell cycle distribution. Cells were trypsinized and collected at different incubation timepoints and stored at −20 °C with 70% ethanol for at least 24 h. Then ethanol was removed and the cells were washed with PBS and stained for 30 min with 500 µL of a solution containing 0.001% triton, 0.2% *w*/*v* RNase and 0.02% PI.

## 4. Conclusions

To our knowledge, we have successfully developed the first DA reaction of thiophene at atmospheric pressure and room temperature. The use of AlCl_3_ as a catalyst provides an accessible, economic and easy way to obtain new maleimido–thiophene DA adducts with good yields. We have also determined optimal conditions for this reaction. An equimolar ratio of AlCl_3_ and maleimide, excess of thiophene and DCM as solvent provides yields in the range of 40% after 96 h at room temperature. This method is also stereospecific, leading the reaction to the preferable exo form. The use of more equivalents of AlCl_3_ pushes the reaction to the formation of 3-thiophen succinimides in a Friedel–Crafts-type reaction. AlCl_3_ is also able to catalyze DA reaction of selenophene, but this adducts are not stable and a chelotropic extrusion of selenium leads to new dienes **9**–**11,** followed by a second DA with maleimide to obtain butterfly-like diazatetradecenes **12**–**14**. The introduction of a selenocyanate group in a thiophene DA adduct generates a new analog (compound **15**) with excellent cytotoxic profile in the 60 cell lines tested by the NCI, achieving an average GI_50_ value of 1.98 µM. Moreover, this compound induces cell cycle arrest at the G2/M phase of the cell cycle on HT-29 cells. These results can pave the road for new bioactive thiophene DA adducts, a barely studied field.

## Figures and Tables

**Table 1:** Experimental conditions tested in the thiophene DA reaction. Effects of solvent, temperature, reaction time and reagents’ proportions in the reaction were optimized.

Entry	Solvent	(1):(2)	eq AlCl_3_	Temp	Time (h)	Yield (%) ^1^ (3)	Yield (%) ^1^ (4)	(3):(4)	Yield (%) ^1^ (5)
1	DCM	1:1	0.5	RT	24	4.4	1.1	78:22	-
2	DCM	1:1	1	RT	24	10.5	2.4	82:18	2.0
3	DCM	1:1	2	RT	24	11.8	0.9	94:6	21.7
4	DCM	5:1	0.5	RT	24	11.0	2.7	80:20	2.7
5	DCM	5:1	1	RT	24	25.9	7.4	77:23	4.6
6	DCM	5:1	2	RT	24	-	-	-	31.0
7	DCM	5:1	1	RT	48	33.4	6.8	83:17	4.8
8	DCM	5:1	1	RT	72	37.3	3.9	90:10	11.6
9	DCM	5:1	1	RT	96	47.2	4.9	91:9	8.8
10	DCM	5:1	1	Reflux	8	15.4	2.1	87:13	2.1
11	DCM	5:1	1	Reflux	24	38.8	3.0	93:7	6.7
12	DCM	5:1	1	Reflux	48	8.2	0.6	93:7	-
13	THF	5:1	1	RT	24	-	-	-	-
14	Et_2_O	5:1	1	RT	24	-	-	-	-
15	CHCl_3_	5:1	1	RT	24	8	3.2	72:28	12.1
16	DCM	1:1	0.5	RT	24	4.4	1.1	78:22	-
17	DCM	1:1	1	RT	24	10.5	2.4	82:18	2.0
18	DCM	1:1	2	RT	24	11.8	0.9	94:6	21.7

^1^ Yields were calculated from the crude reaction by q-NMR using dimethyl sulfone as internal standard. 1H-NMR (CDCl_3_) signals chosen: exo **3** (t, 6.67 ppm, 2H); endo **4** (t 6.61 ppm, 2H); Friedel–Crafts-type byproduct **5** (dd. 3.47 ppm, 1H); dimetylsulfone (s, 3.00 ppm, 6H).

**Table 2:** Effects of Lewis acids in the DA reaction.

Entry	Lewis Acid	Yield (%) ^1^ 3	Yield (%) ^1^ 4	(3):(4)	Yield (%) ^1^ 5
5	AlCl_3_	25.9	7.4	77:23	4.6
16	Me_2_AlCl	0.05	0.2	66:34	0
17	FeCl_3_	0	0	-	0
18	SnCl_4_	0	0	-	0
19	TiCl_4_	0.019	0.002	81:19	0
20	BF_3_·H_2_0	0	0	-	0

^1^ Yields were calculated from the crude reaction by q-NMR using dimethyl sulfone as internal standard. ^1^H-NMR (CDCl_3_) signals chosen: exo **3** (t, 6.67 ppm, 2H); 2H; endo **4** (t 6.61 ppm, 2H); Friedel–Crafts-type byproduct **5** (dd. 3.47 ppm, 1H); dimetylsulfone (s, 3.00 ppm, 6H). The conditions used for the reaction include 1 eq. of the corresponding Lewis acid, 5 eq. of thiophene and DCM as solvent at room temperature for 24 h.

**Table 3:** Formation of DA adducts with different dienophiles. Endo:exo ratio along with crude and isolated yields for the different adducts obtained.

Dienophile	Adduct	Endo:exo ^a^	Yield (%) exo ^a^	Yield (%) endo ^a^	Isolated Yield (%) exo
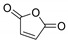	No	n.d.	n.d.	n.d.	n.d.
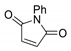	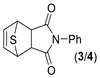	9:91	50	4.9	33
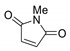	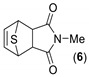	31:69	32	15	25
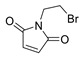	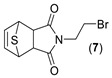	10:90	42	3.8	36
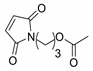	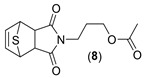	19:81	10	2.4	7.1
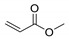	No	n.d.	n.d.	n.d.	n.d.
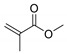	No	n.d.	n.d.	n.d.	n.d.
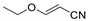	No	n.d.	n.d.	n.d.	n.d.

^a^ Yields were calculated from the crude reaction by q-NMR using dimethyl sulfone as an internal standard. ^1^H-NMR (CDCl_3_) signals chosen: exo **3** (t, 6.67 ppm, 2H); endo **4** (t, 6.61 ppm, 2H); Friedel–Crafts-type byproduct **5** (dd. 3.47 ppm, 1H); dimetylsulfone (s, 3.00 ppm, 6H). (n.d.) not detected. The conditions used for the reaction include 1 eq. of AlCl_3_, 5 eq. of thiophene and DCM as solvent at room temperature for 24 h.

## Data Availability

Not applicable.

## Supplementary Materials

The following supporting information can be downloaded online.

## Sample Availability

Samples of the compounds are available from the authors.
