# Supplementary material for: New Experimental Conditions for Diels–Alder and Friedel-Crafts Alquilation Reactions with Thiophene: A New Selenocyanate with Potent Activity against Cancer
Source: Molecules. 2022 Feb 1;27(3):982. doi: 10.3390/molecules27030982 (PMC8839041; doi:10.3390/molecules27030982)
Supplement: Supplementary file 1 [file molecules-27-00982-s001.zip › molecules-1544570-supplementary.pdf]

molecules

MDPI

Article (Supplementary Information)

New experimental conditions for Diels-Alder and Friedel-Crafts Alquilation Reactions with thiophene: A new selenocyanate with potent activity against cancer

Gorka Calvo-Martín [1], Daniel Plano [1, 2,*] and Carmen Sanmartín [1,2,*]

[1] Facultad de Farmacia y Nutrición, Departamento de Tecnología y Química Farmacéuticas. Universidad de Navarra. Irunlarrea 1, E-31008 Pamplona, Spain; gcalvo.3@alumni.unav.es (GC-M); dplano@unav.es (DP); sanmartin@unav.es (CS)

[2] Instituto de Investigación Sanitaria de Navarra (IdiSNA) Irunlarrea 3, E-31008 Pamplona Spain

* Correspondence: dplano@unav.es (DP); sanmartin@unav.es (CS); Tel.: (optional; include country code; if there are multiple corresponding authors, add author initials)

Abstract: The reactivity of thiophene in Diels-Alder reactions is investigated with different maleimide derivatives. In this paper, we have synthesized for the first time the Diels-Alder adducts of thiophene at room temperature and atmospheric pressure. Maleimido-thiophene adducts were promoted by $AlCl_3$. The effects of solvent, time, temperature and the use of different Lewis acids were studied, showing dramatic effects for solvent and Lewis acid. Furthermore, the catalysis with $AlCl_3$ is highly stereoselective, providing preferably the exo form of the adduct. Additionally, we also discovered the ability of $AlCl_3$ to catalyze the arylation of maleimides to yield 3-aryl succinimides in a straightforward manner following a Michael-type addition. The inclusion of a selenocyanate group contributes to the cytotoxic activity of the adduct. This derivatization (from compound **7** to compound **15**) results in an average GI_{50} value of 1.98 μM in the DTP (NCI-60) cell panel, resulting especially active in renal cancer cells.

Keywords: Thiophene; Selenophene; Diels-Alder; Cycloadition; Michael reaction; Selenium; Cancer

Citation: Calvo-Martín, G.; Plano, D.; Sanmartín, C. New Experimental Conditions for Diels–Alder and Friedel-Crafts Alquilation Reactions with Thiophene: A New Selenocyanate with Potent Activity against Cancer. *Molecules* **2022**, *27*, 982. https://doi.org/10.3390/molecules27030982

Academic Editor: M. Amparo F. Faustino

Received: 22 December 2021
Accepted: 29 January 2022
Published: 1 February 2022

Publisher's Note: MDPI stays neutral with regard to jurisdictional claims in published maps and institutional affil-iations.

Copyright: © 2022 by the authors. Submitted for possible open access publication under the terms and conditions of the Creative Commons Attribution (CC BY) license (https://creativecommons.org/licenses/by/4.0/).

Table of content

NMR spectra for newly synthesized final compounds……………………………………………………….Figures S1-S19
NMR spectra for intermediates…………………………………………………………………......……….Figures S20-S27
Yields calculations by q-NMR……..…………………………………………………………………..……….Figure S28-S33
HRMS spectra for newly synthesized compounds……………………………………………..…………….Figures S34
NCI´s DTP dose-response report for compound **15** …………………………………………………………..Figures S35-S38

1. NMR spectra for newly synthesized final compounds.

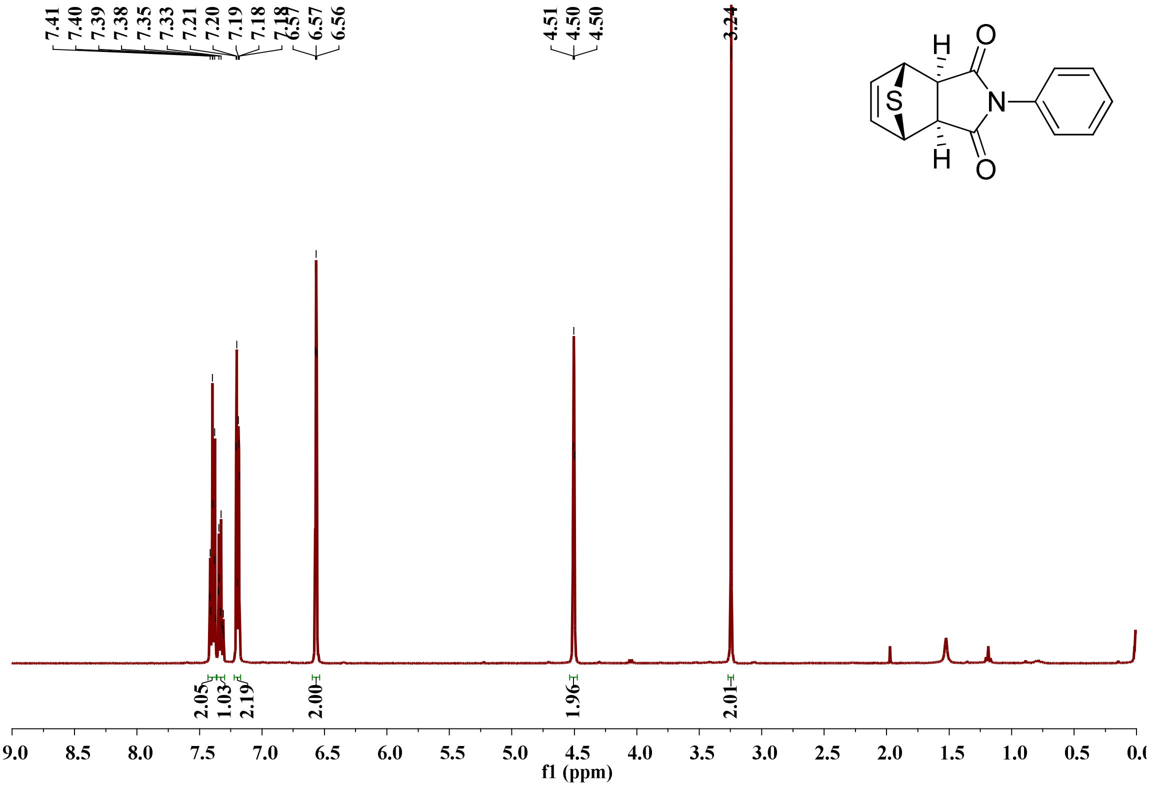

Figure S1. **¹H-NMR of compound 3.**

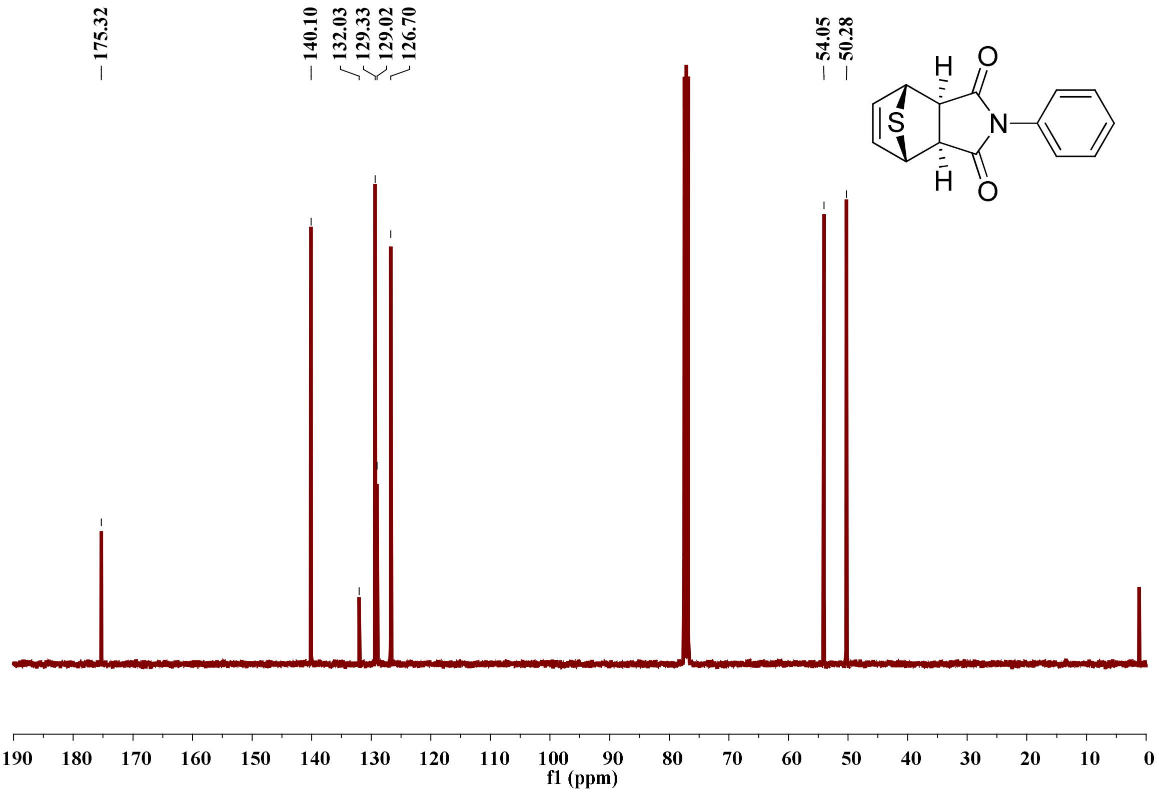

Figure S2. **¹³C-NMR of compound 3.**

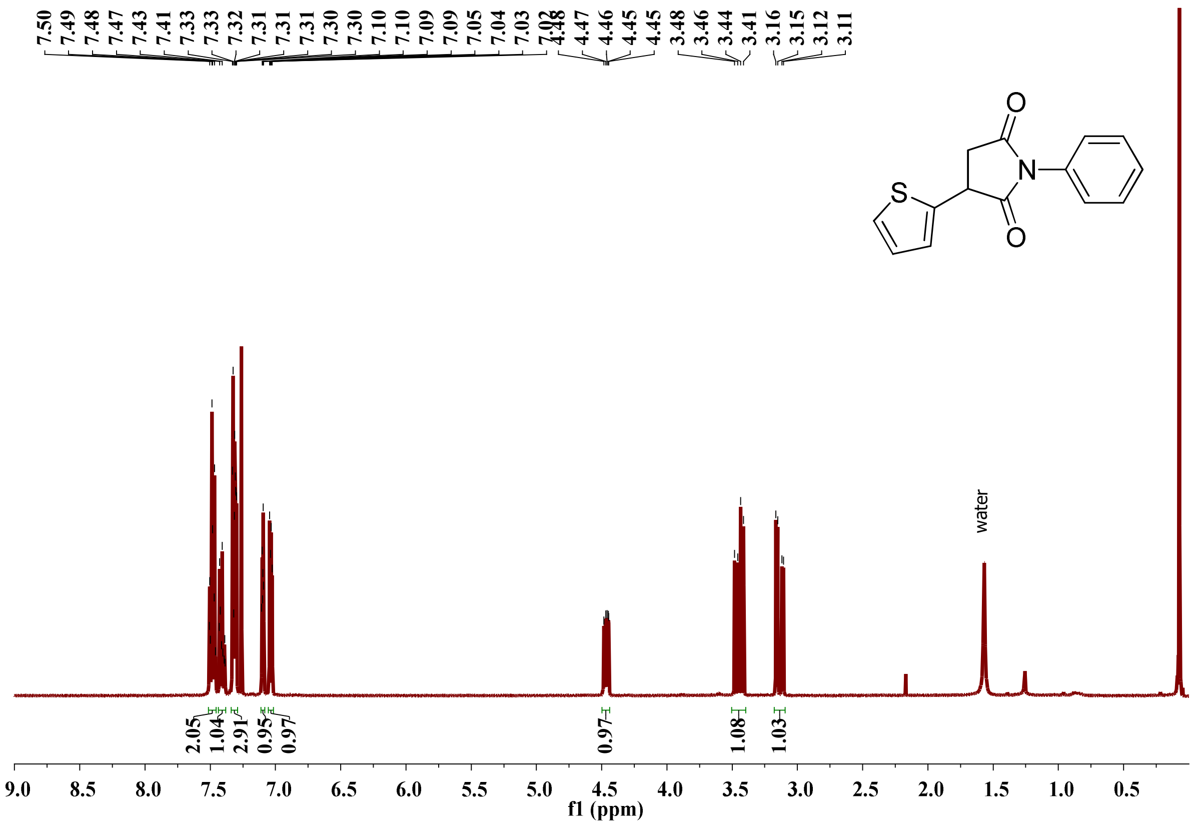

Figure S3. ^{1}**H-NMR of compound 5.**

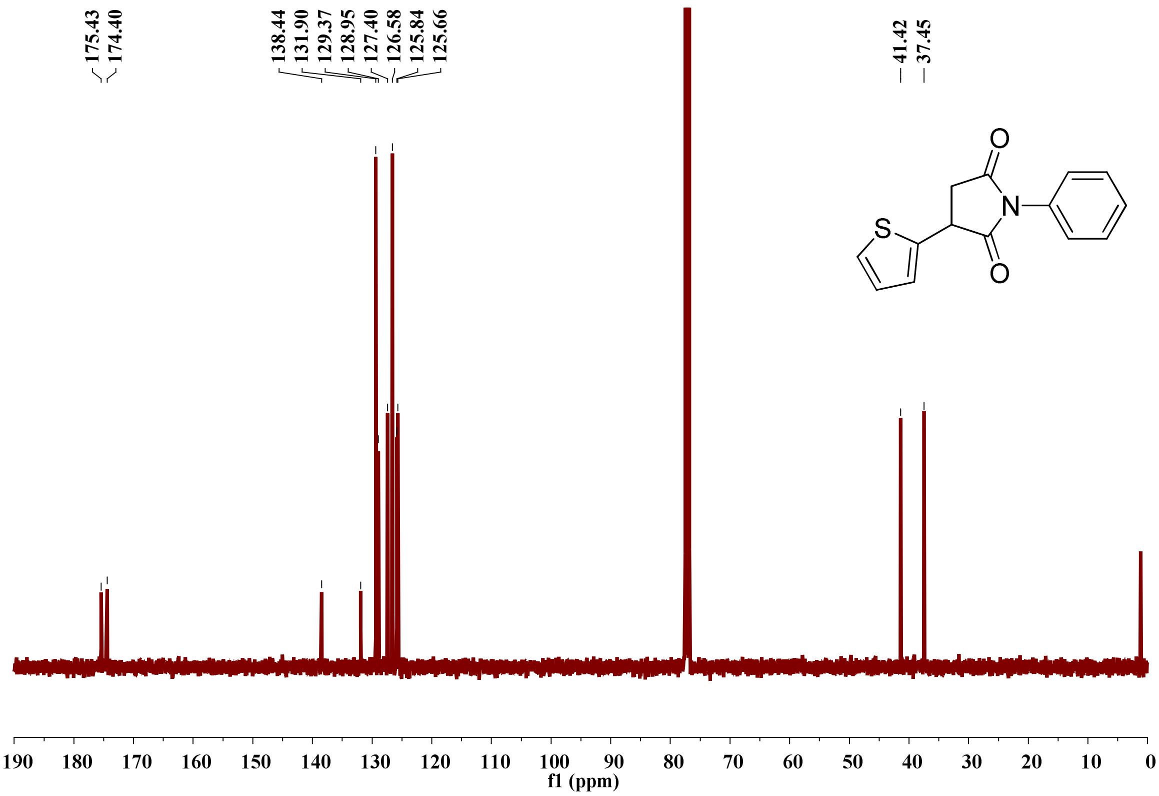

Figure S4. ^{13}**C-NMR of compound 5.**

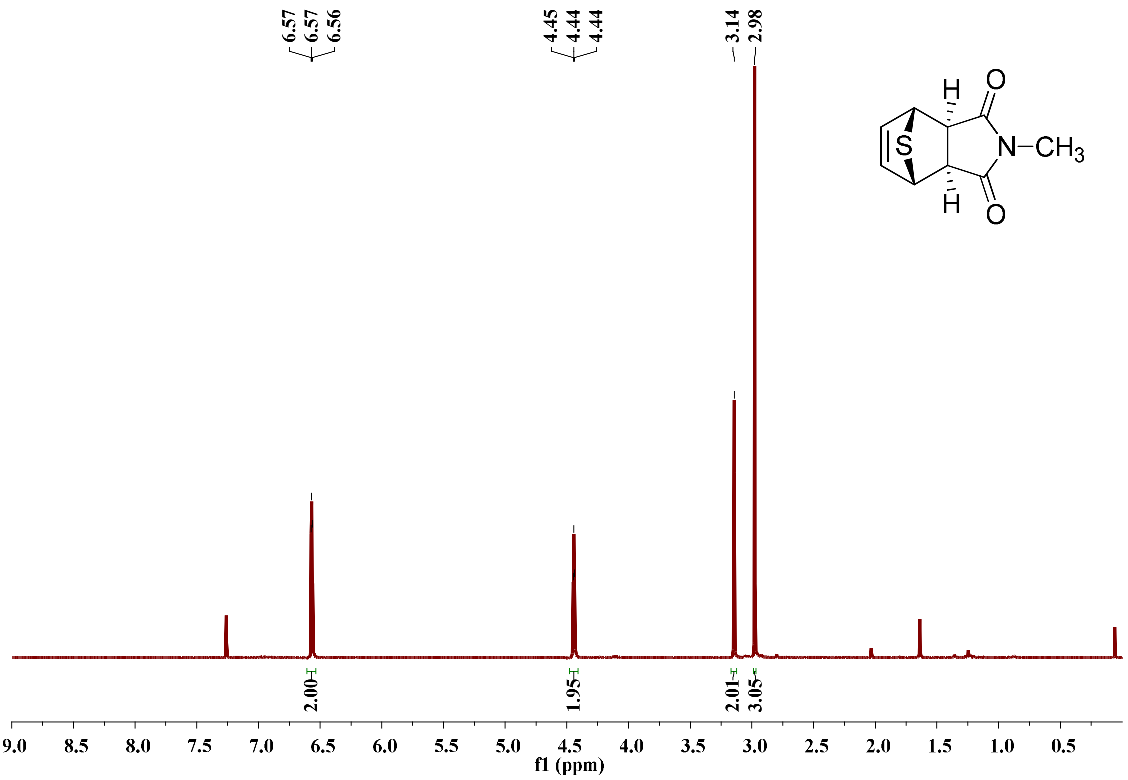

Figure S5. ^{1}**H-NMR of compound 6.**

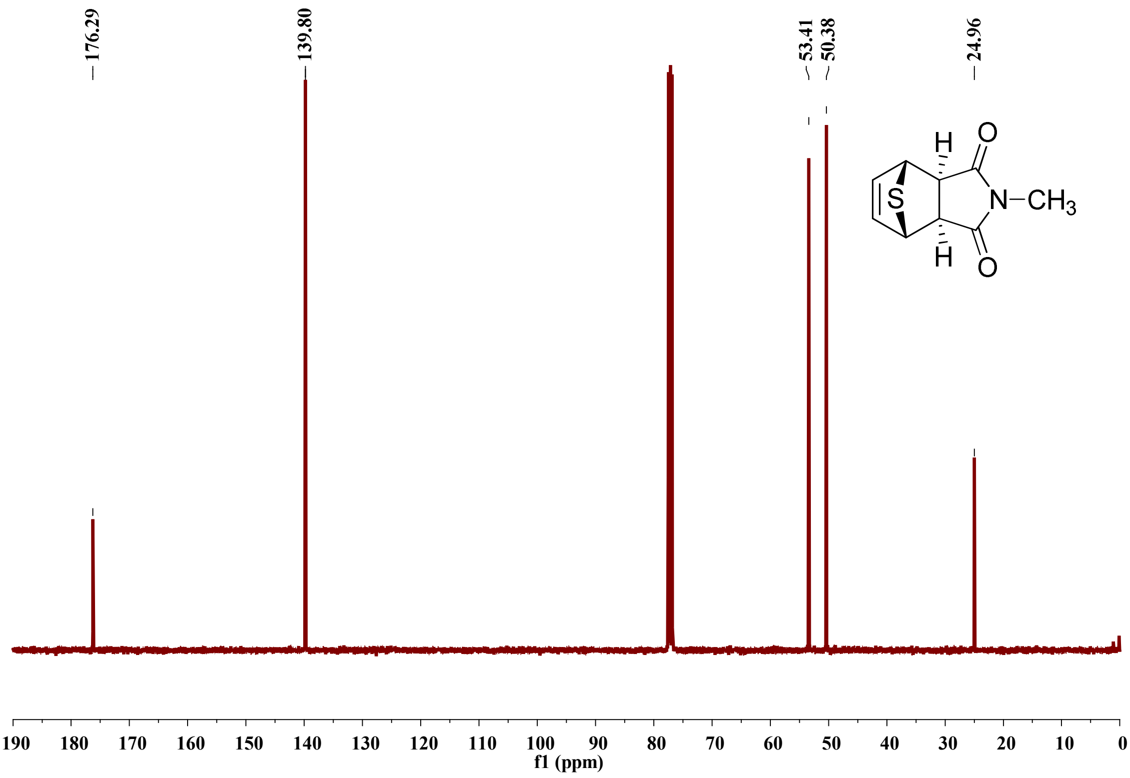

Figure S6. ^{13}**C-NMR of compound 6.**

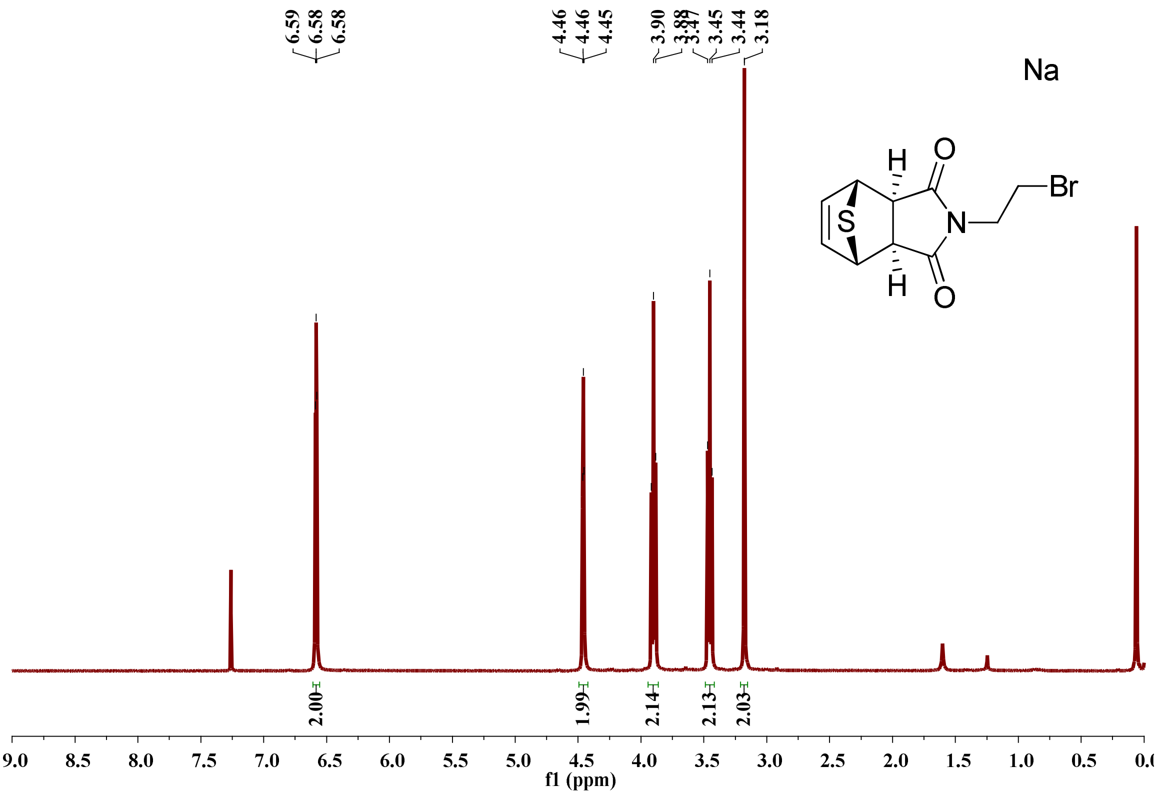

Figure S7. ¹H-NMR of compound **7**.

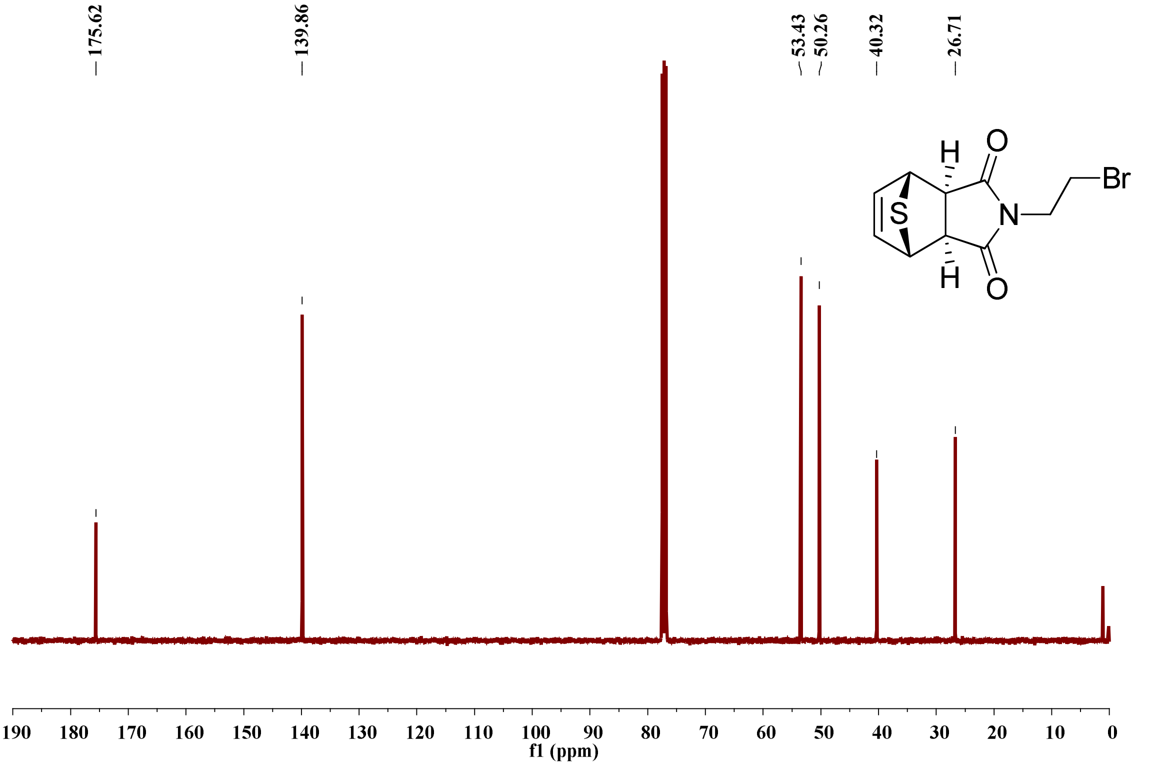

Figure S8. ¹³C-NMR of compound **7**.

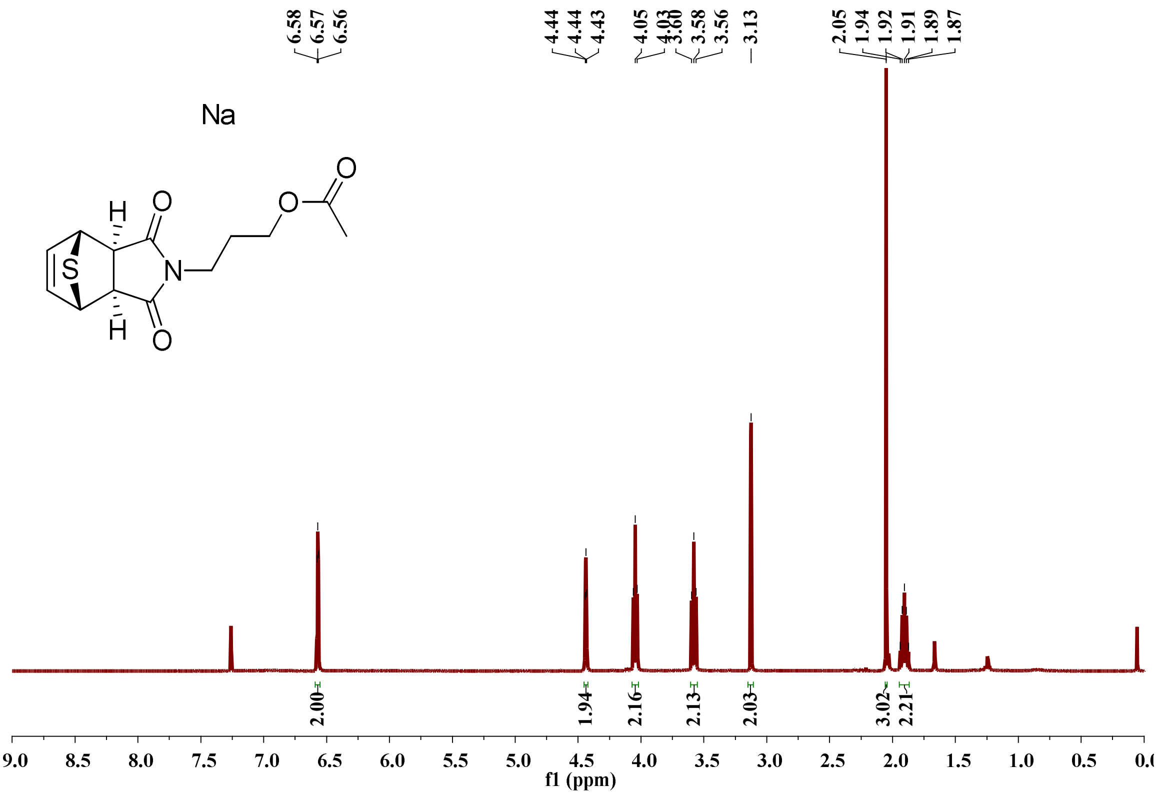

Figure S9. ^{1}H-NMR of compound 8.

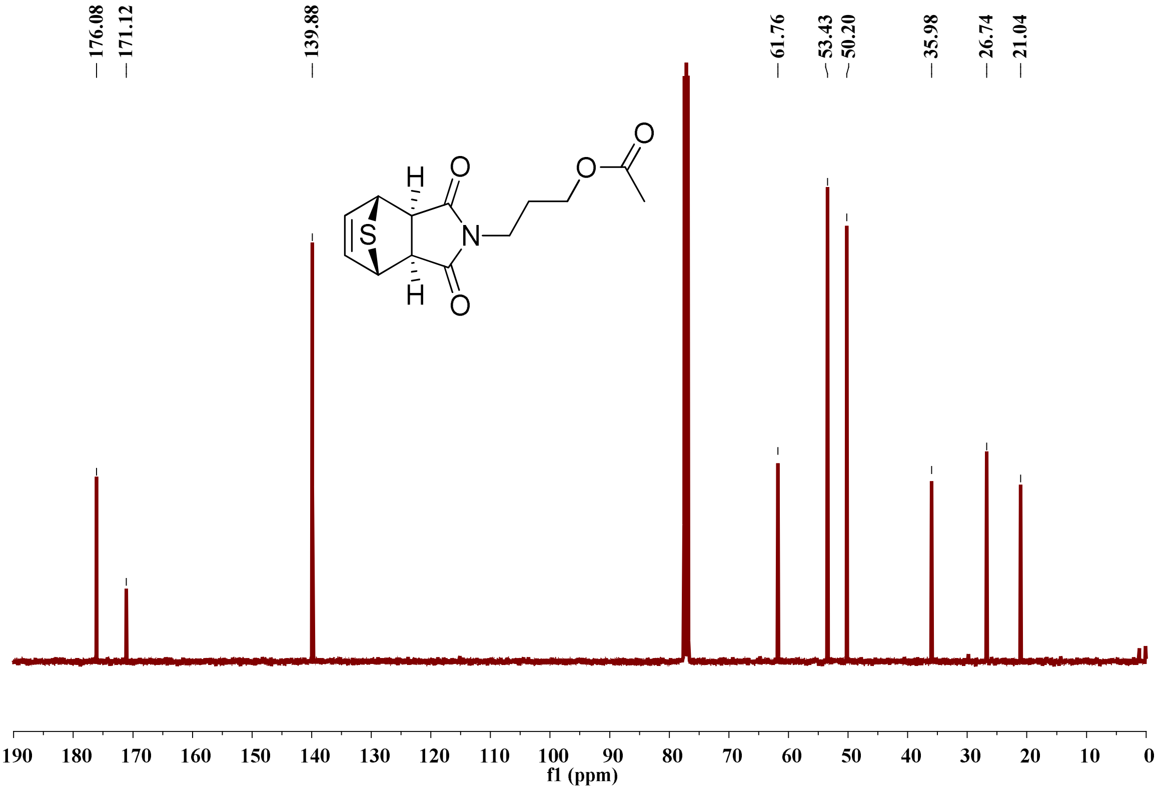

Figure S10. ^{13}C-NMR of compound 8.

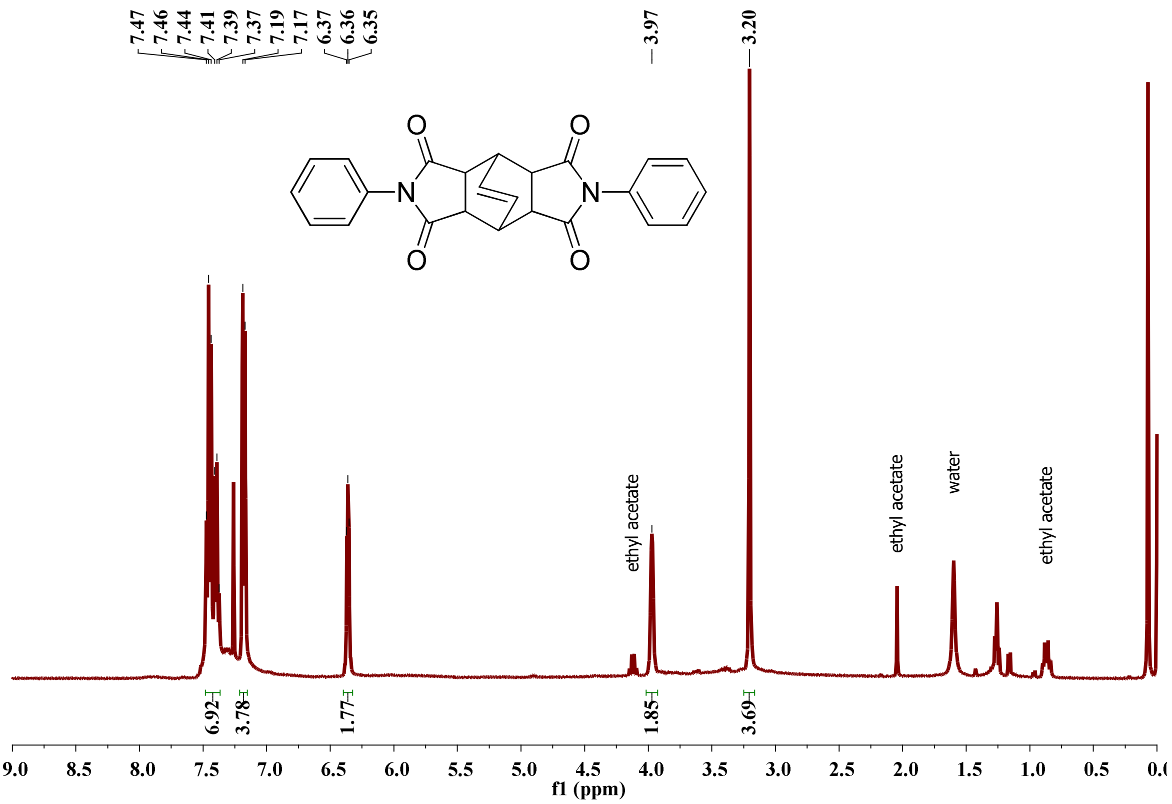

Figure S11. ^{1}H-NMR of compound 12.

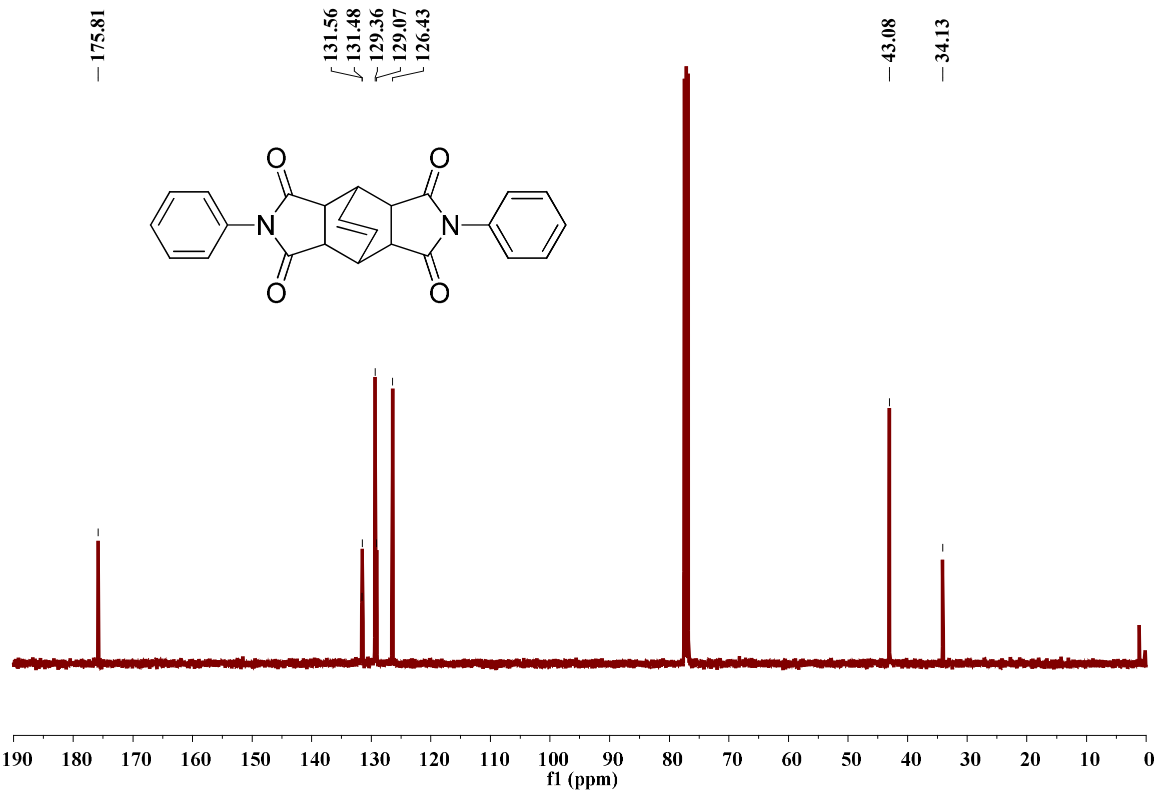

Figure S12. ^{13}C-NMR of compound 12.

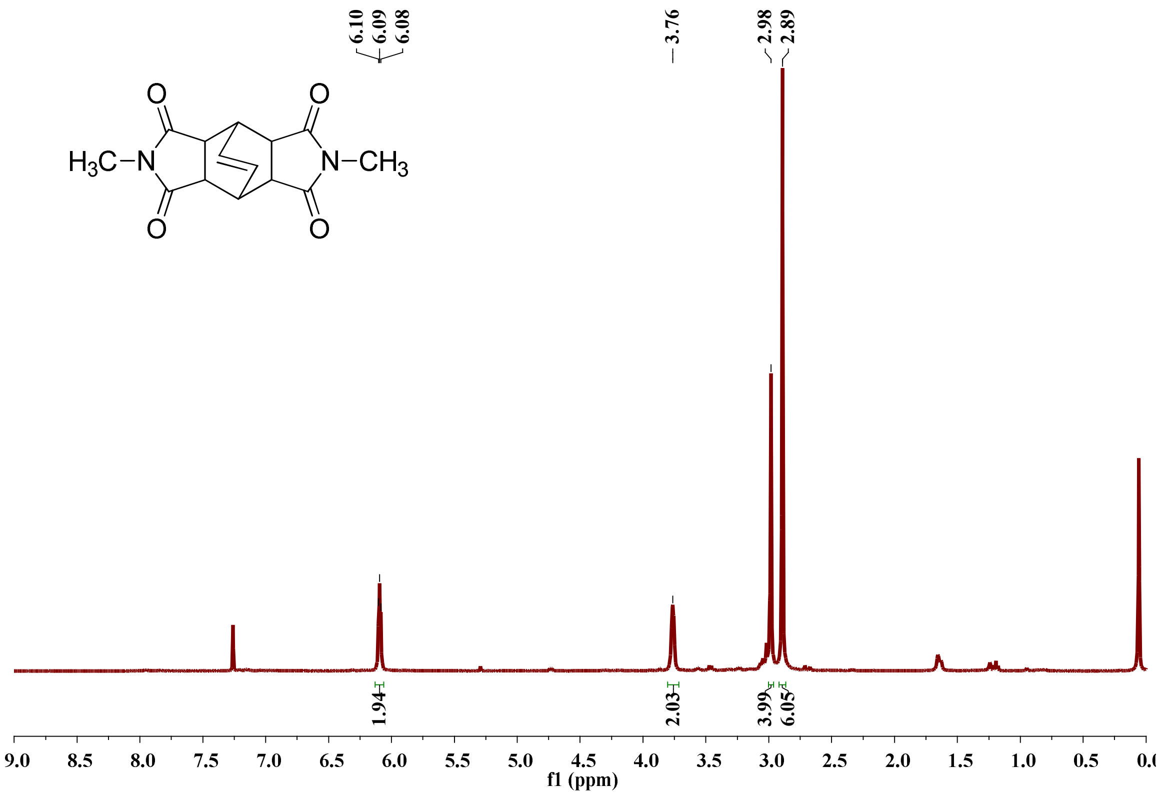

Figure S13. **^{1}H-NMR of compound 13.**

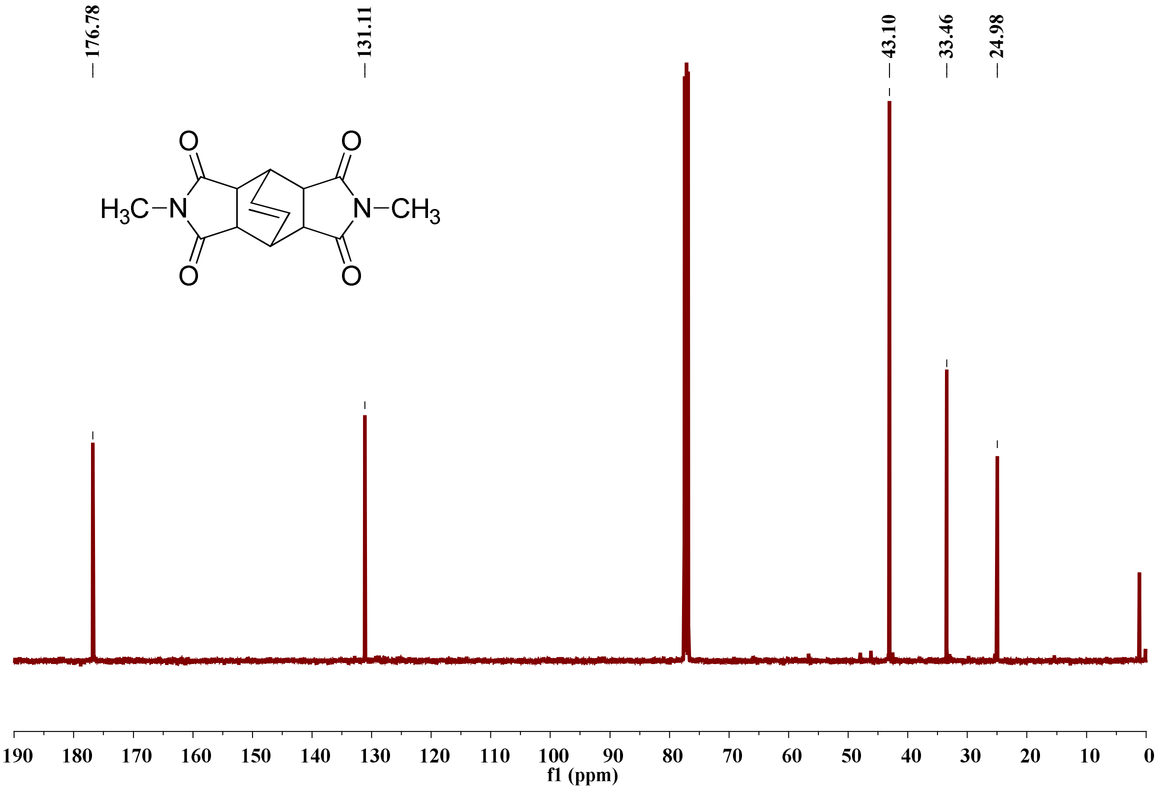

Figure S14. **^{13}C-NMR of compound 13.**

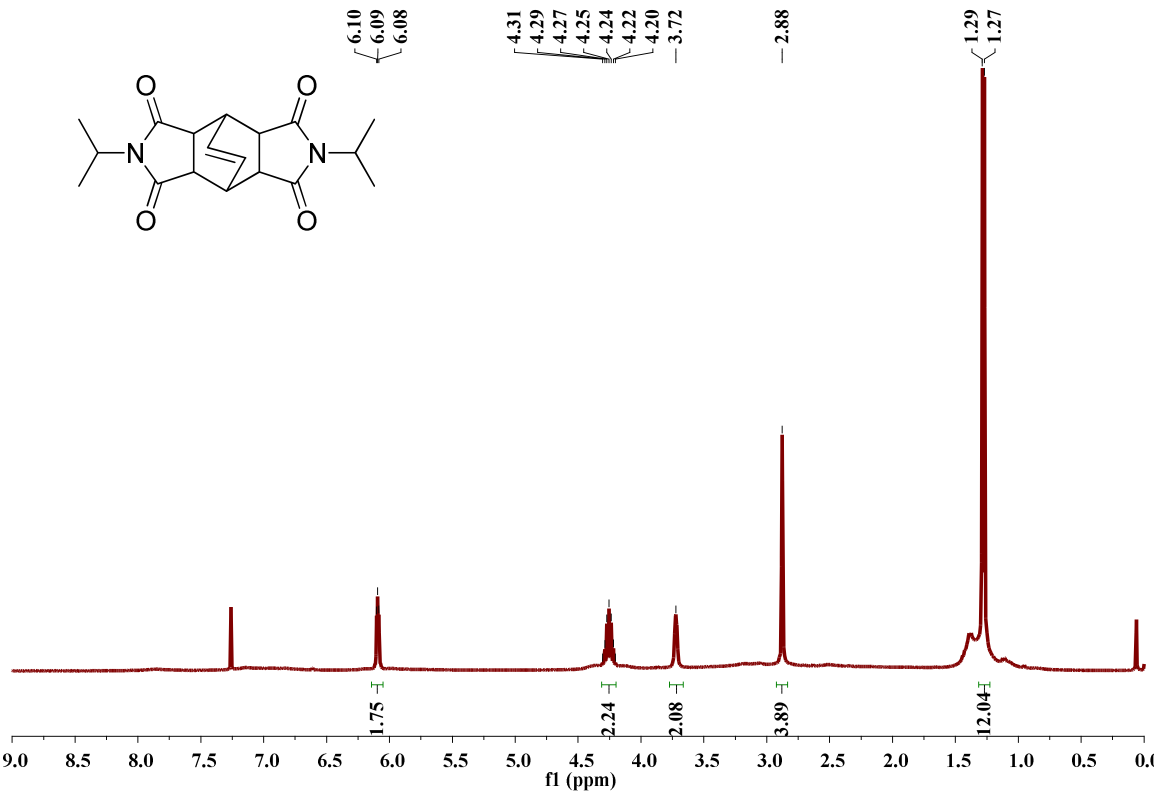

Figure S15. **^{1}H-NMR of compound 14.**

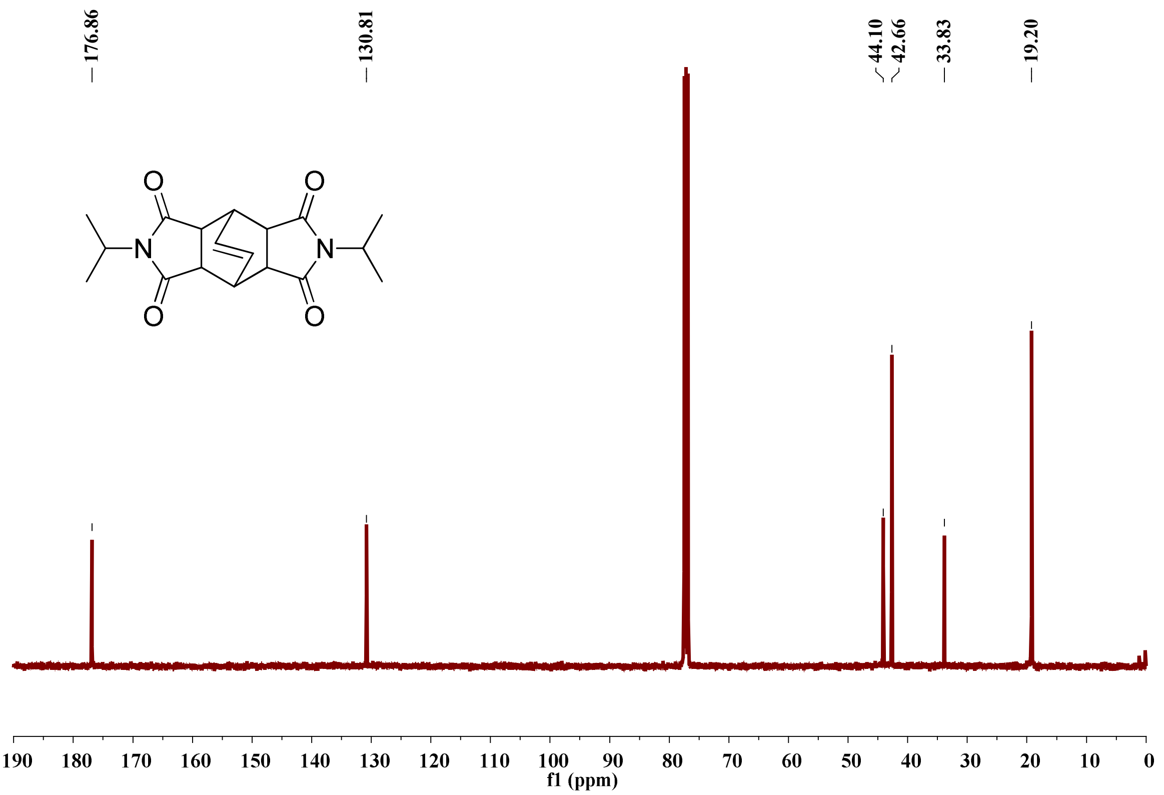

Figure S16. **^{13}C-NMR of compound 14.**

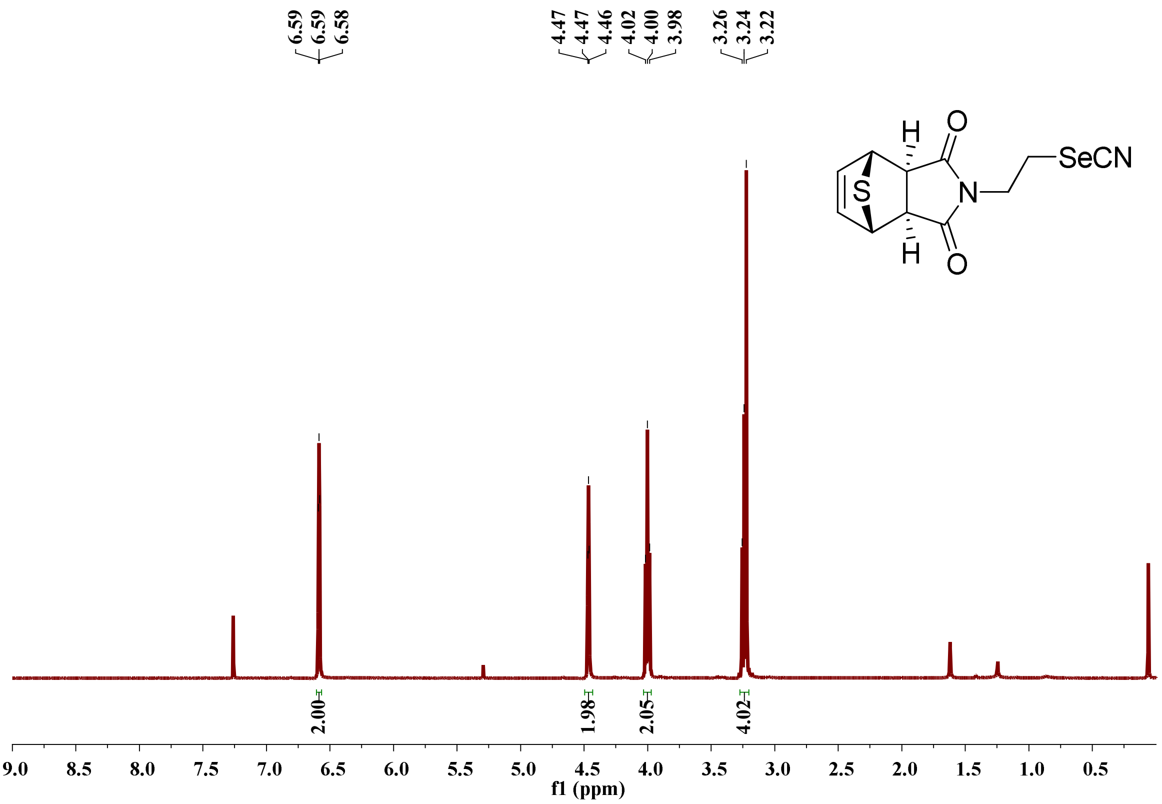

Figure S17. ^{1}**H-NMR of compound 15.**

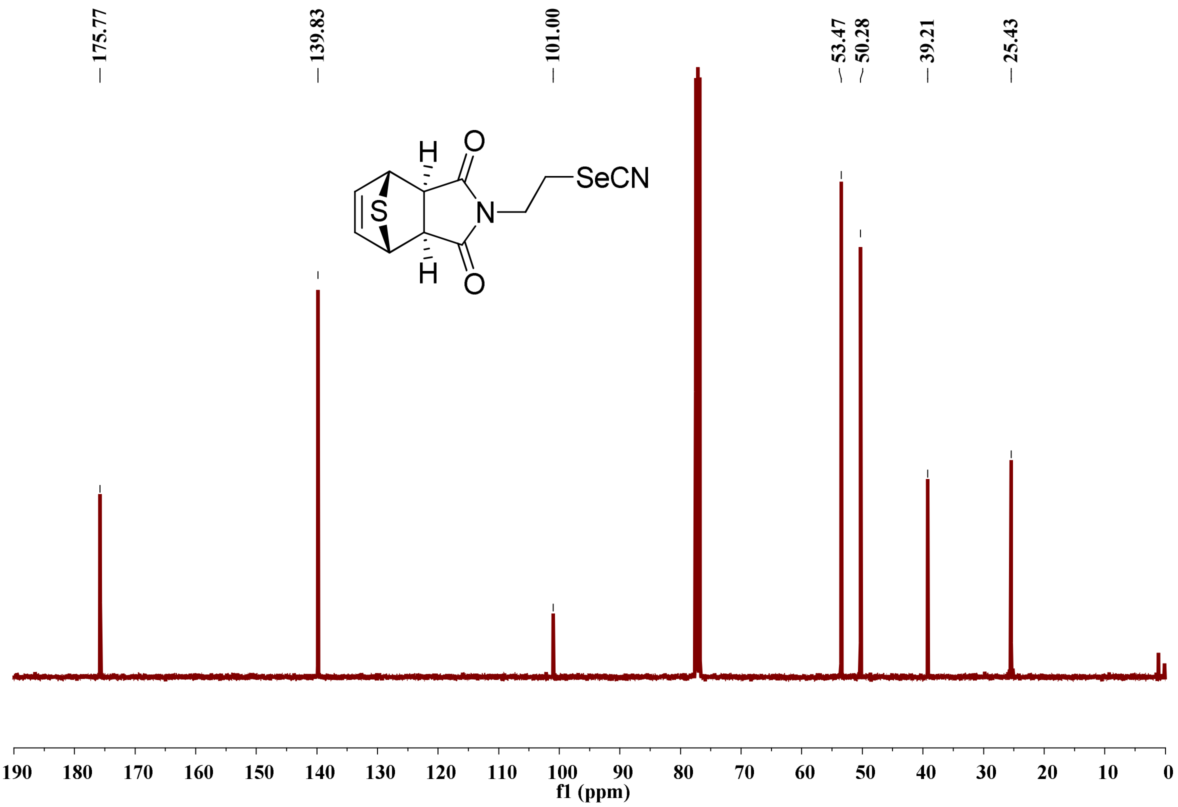

Figure S18. ^{13}**C-NMR of compound 15.**

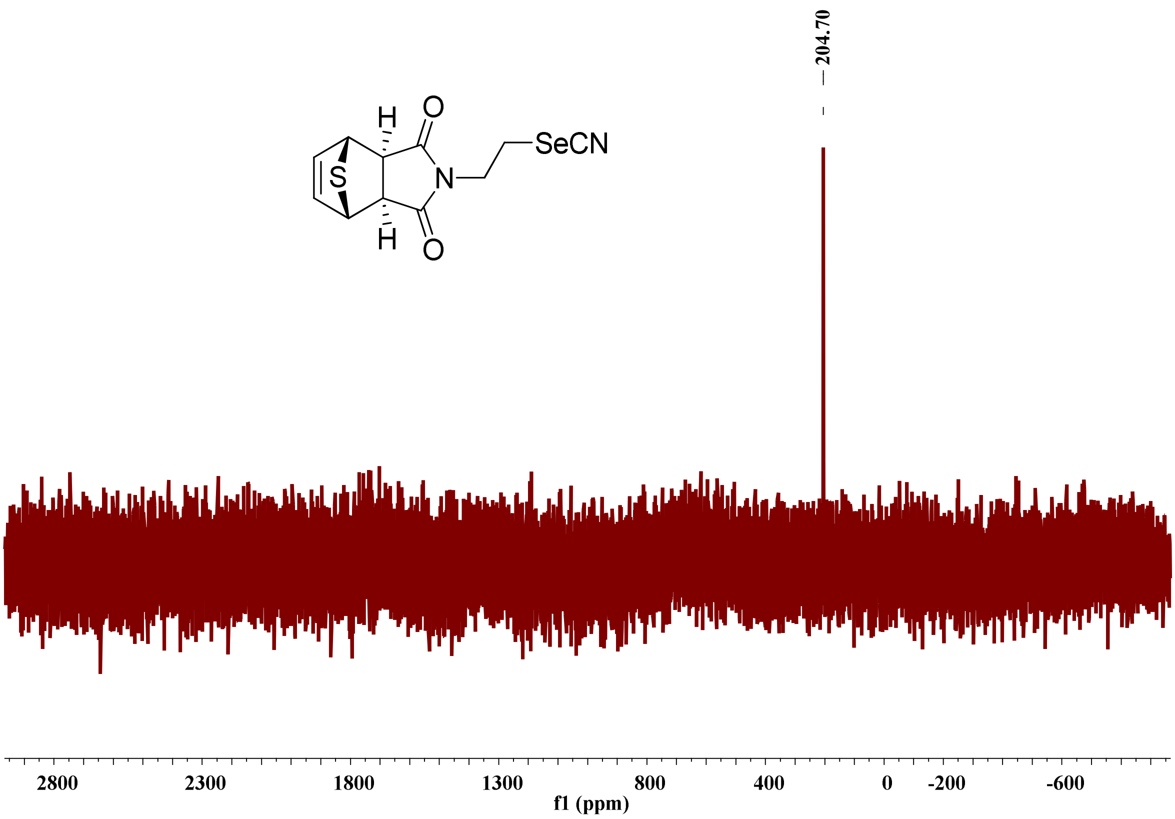

Figure S19. ^{77}Se-NMR of compound 15.

2. NMR spectra for intermediates.

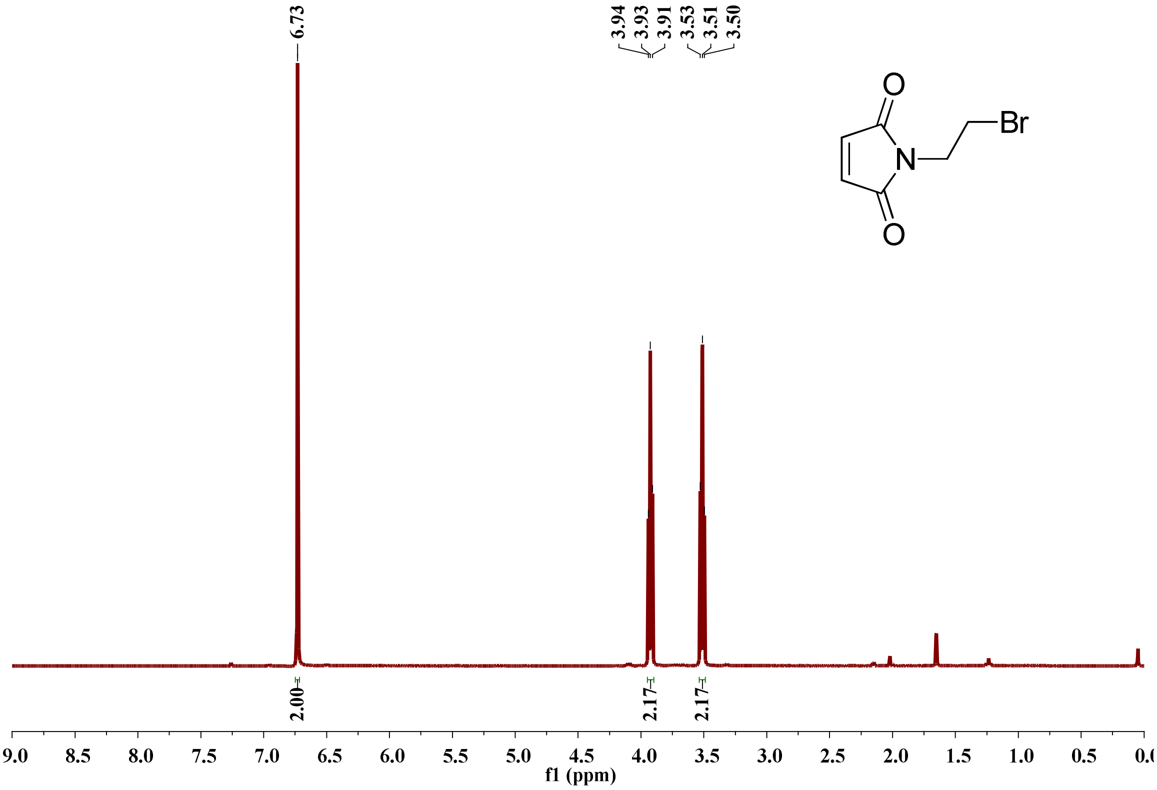

Figure S20. ^{1}H-NMR of *N*-2-bromoethyl maleimide.

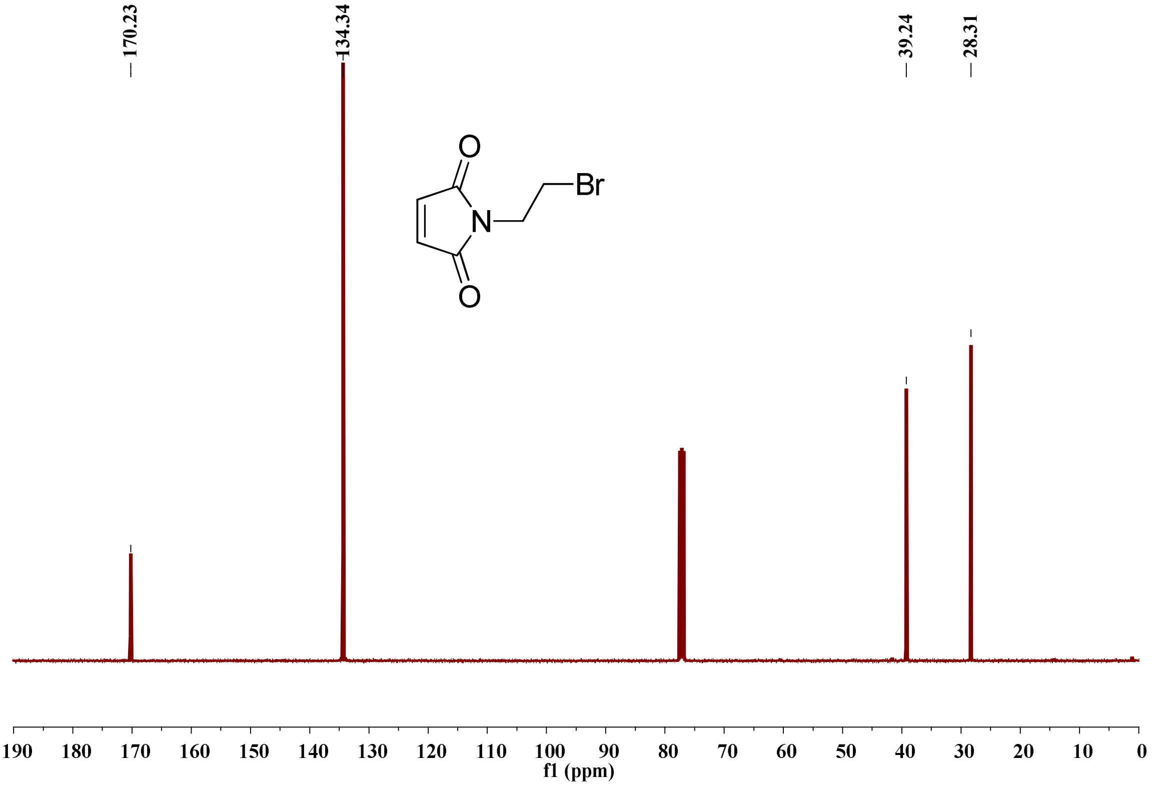

Figure S21. ^{13}C-NMR of *N*-2-bromoethyl maleimide.

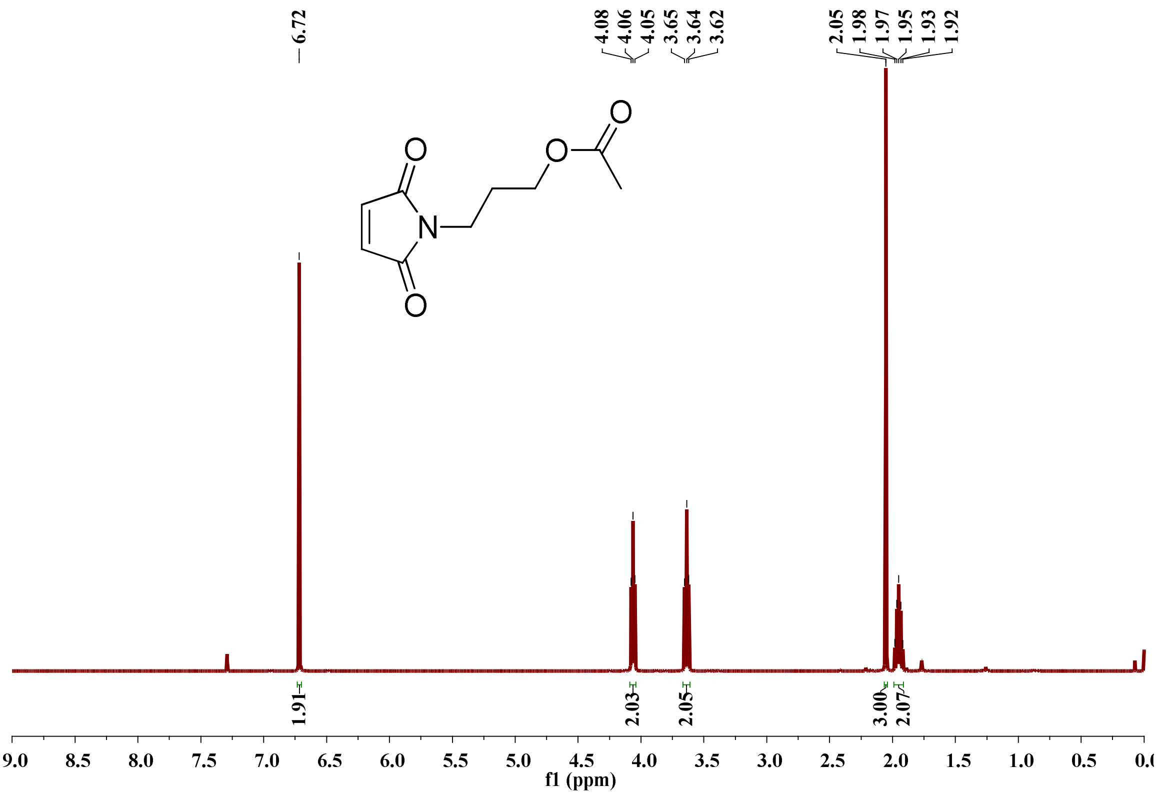

Figure S22. **^{1}H-NMR of *N*-3-acetoxypropyl maleimide.**

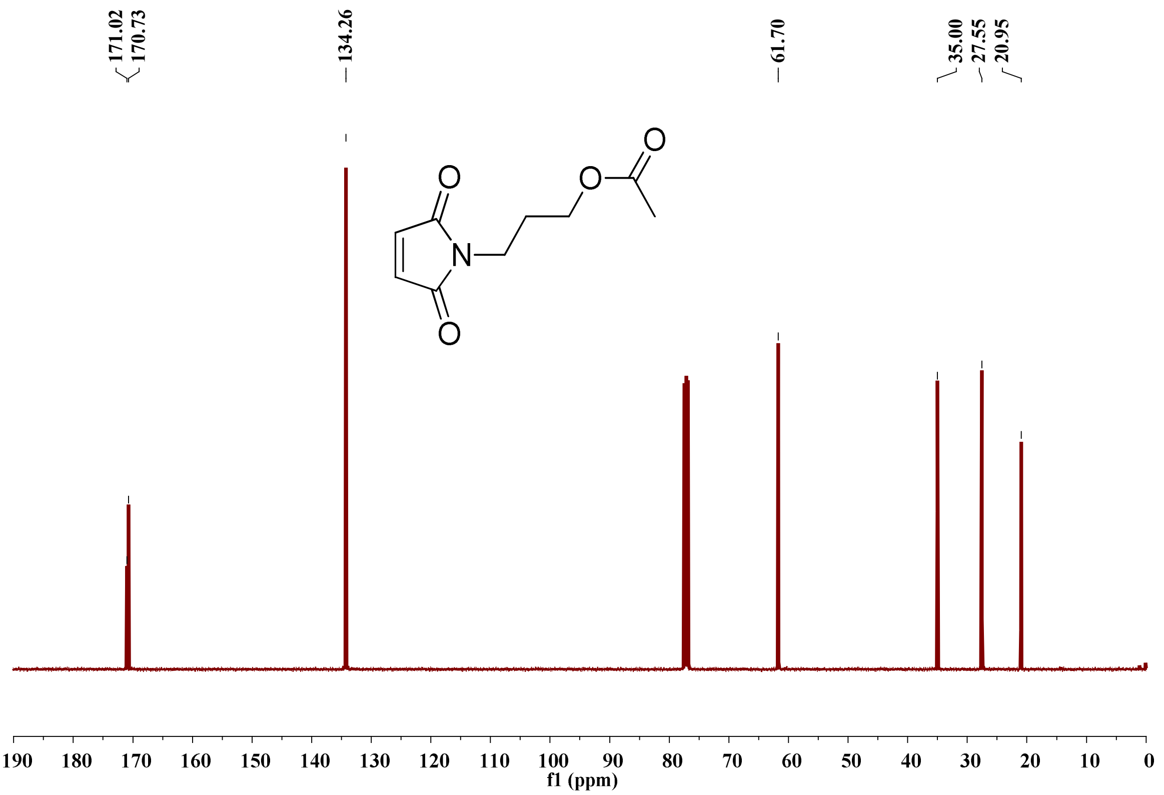

Figure S23. **^{13}C-NMR of *N*-3-acetoxypropyl maleimide.**

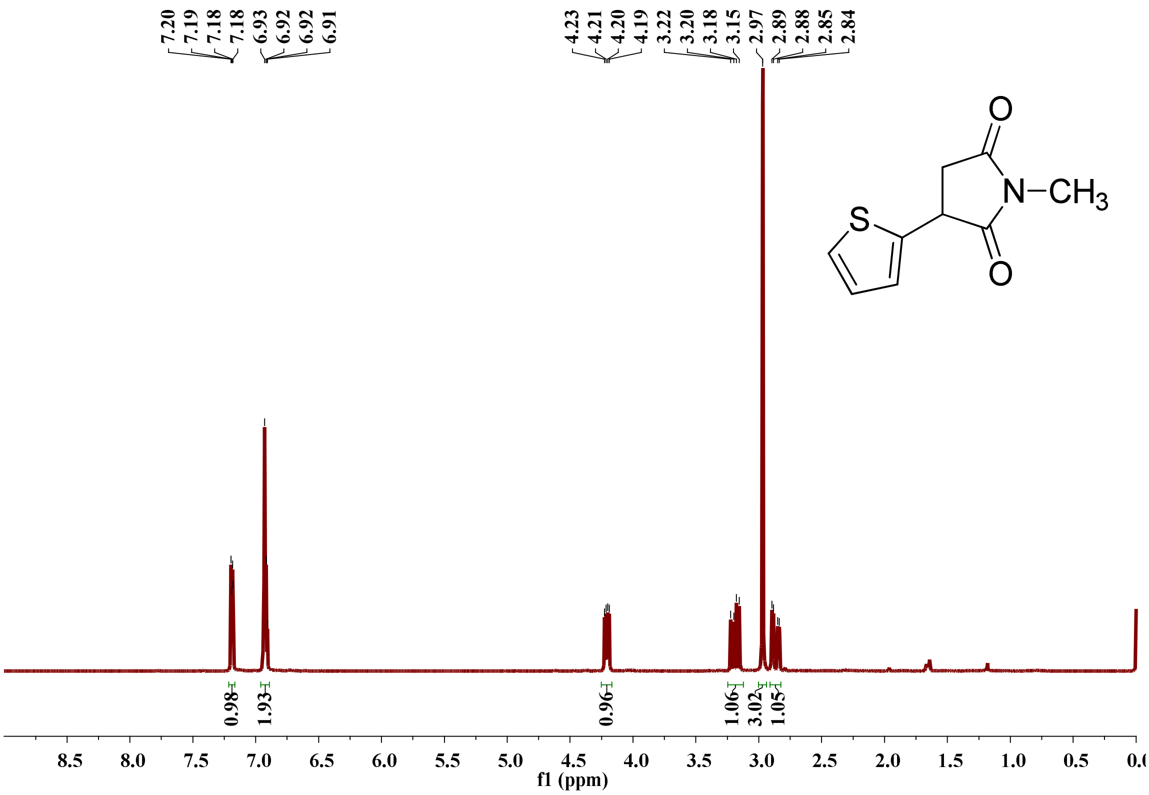

Figure S24. ^{1}H-NMR of 1-methyl-3-(thiophen-2-yl)pyrrolidine-2,5-dione.

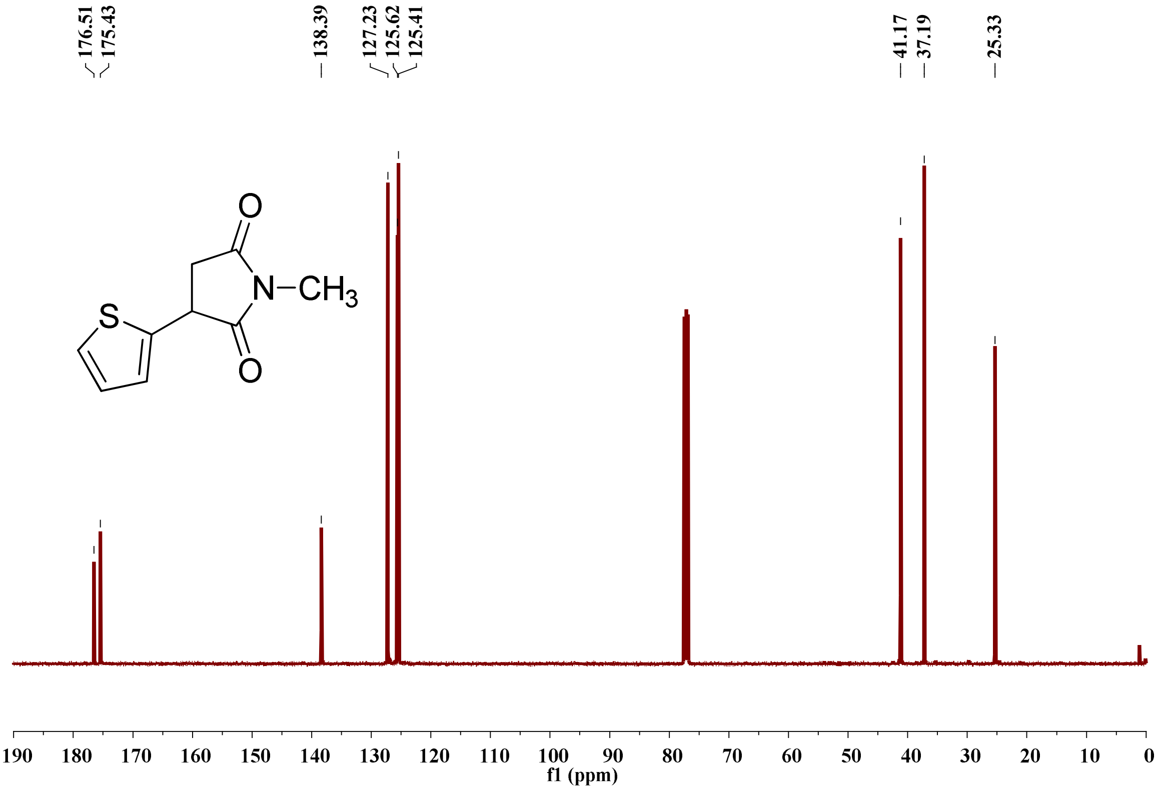

Figure S25. ^{13}C-NMR of 1-methyl-3-(thiophen-2-yl)pyrrolidine-2,5-dione.

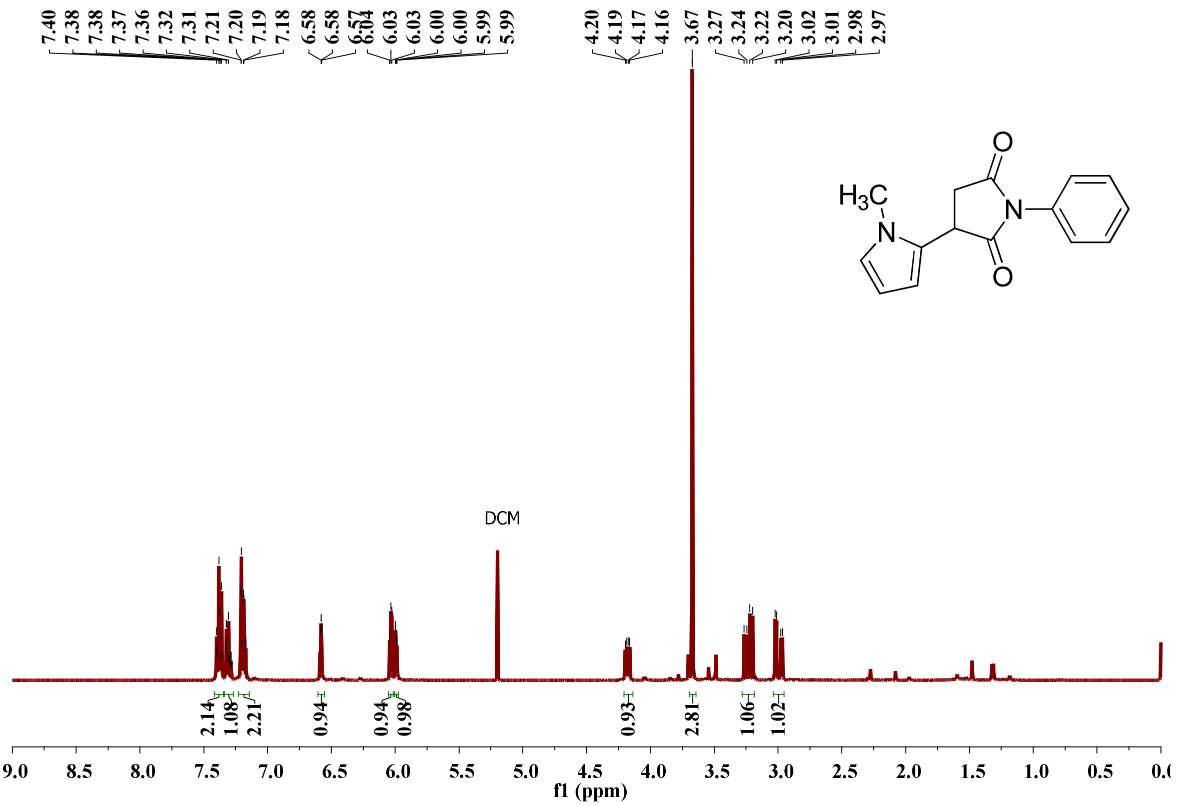

Figure S26. ^{1}H-NMR of 3-(1-methyl-1H-pyrrol-2-yl)-1-phenylpyrrolidine-2,5-dione.

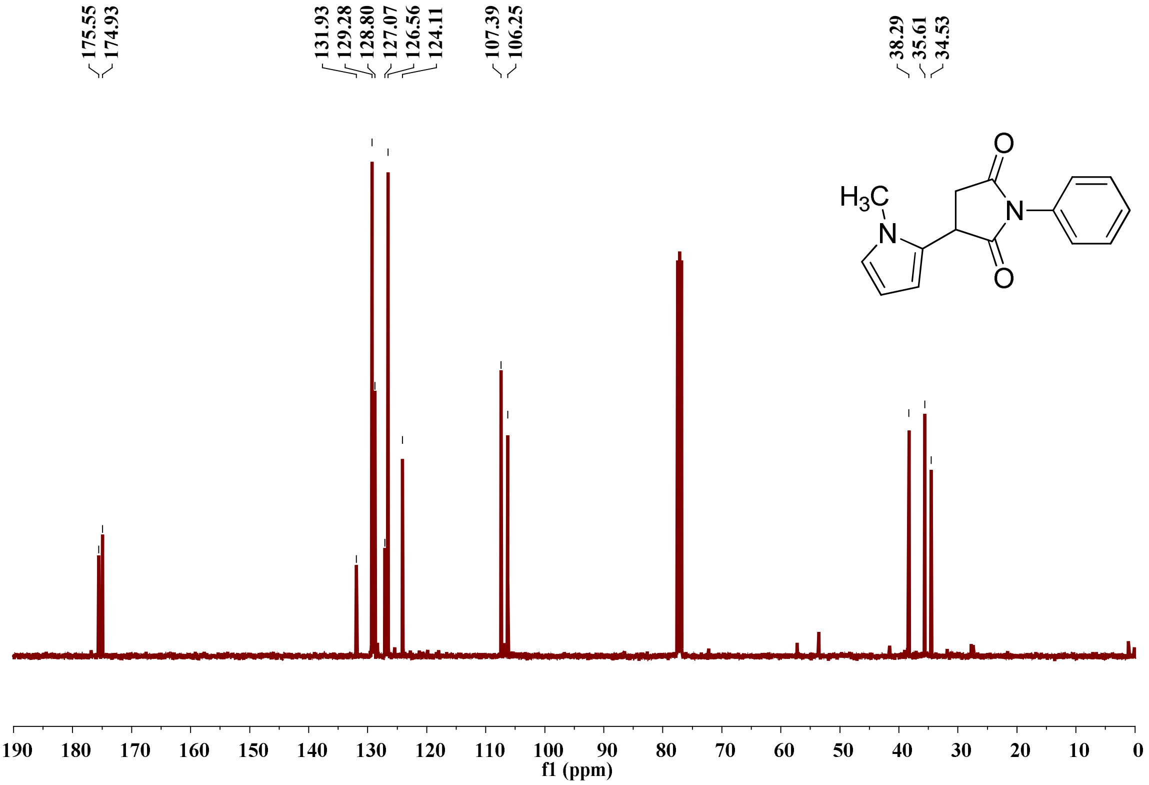

Figure S27. ^{13}C-NMR of 3-(1-methyl-1H-pyrrol-2-yl)-1-phenylpyrrolidine-2,5-dione.

3. Yield calculations by q-NMR.

Yield calculations (entries 1-20) of compounds **3**, **4** and **5** were calculated by q-NMR adding dimethylsulfone tracerCERT® purchased by Sigma-Aldrich as internal standard. A weighed amount of the crude reaction product and a weighed amount of dimethylsulfone were dissolved in 0.5 mL of deuterated chloroform ($CDCl_3$) and placed in a NMR tube. The sample was analyzed by ^{1}H-NMR at 400 mHz as previously described by Kumamoto [1]. ^{1}H NMR signals of the double bound in the *N*-phenylmaleimide–thiophene adducts (endo and exo) and the signal corresponding to the hydrogen attached to the stereogenic center formed in the Michael-type adduct were chosen for the calculations. ^{1}H NMR ($CDCl_3$) signals: exo **3** (t, 6.67 ppm, 2H); endo **4** (t, 6.61 ppm, 2H); Michael-type by-product **5** (dd, 3.47 ppm, 1H) and dimethylsulfone (s, 3.00 ppm, 6H). These signals are presented in Figure S28.

Yield of each compound was calculated as proposed by Sigma-Aldrich® for the tracerCERT® Certified Reference Material using the following formula:

$$Ysample = \frac{I_{Analyte}}{I_{CRM}} \cdot \frac{N_{CRM}}{N_{Analyte}} \cdot \frac{M_{Analyte}}{M_{CRM}} \frac{m_{CRM}}{m_{Sample}} \cdot P_{CRM}$$

Y_{Sample}: yield of the sample as mass fraction
P_{CRM}: purity of the CRM as mass fraction
$I_{Analyte}$: integral of the analyte signal
I_{CRM}: integral of the CRM signal
$N_{Analyte}$: number of analyte protons
N_{CRM}: number of CRM protons
$M_{Analyte}$: molecular mass of the analyte
M_{CRM}: molecular mass of the CRM
m_{Sample}: mass of sample
m_{CRM}: mass of CRM

Reference

1. Kumamoto, K.; Fukada, I.; Kotsuki, H., Diels-Alder reaction of thiophene: dramatic effects of high-pressure/solvent-free conditions. *Angew. Chem. Int. Ed. Engl.* **2004,** 43, 2015-7. 10.1002/anie.200353487.

4. HRMS spectra for newly synthesized compounds.

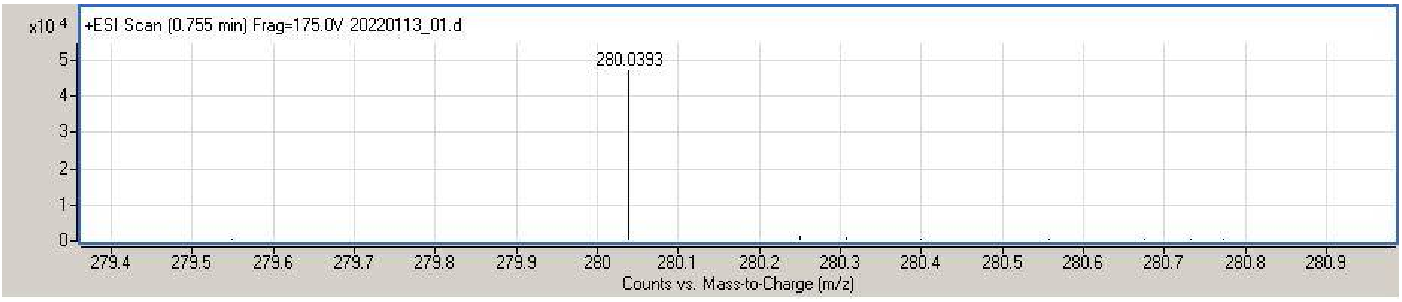

Figure S38. HRMS of compound 3.

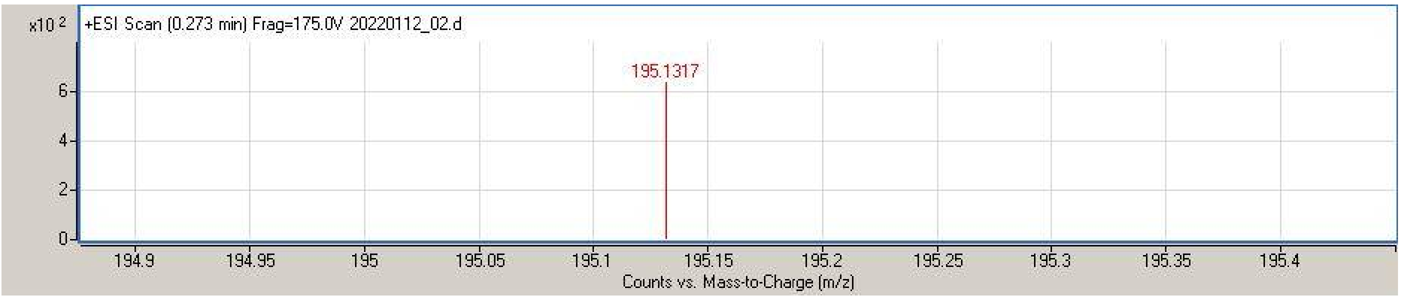

Figure S49. HRMS of compound 6.

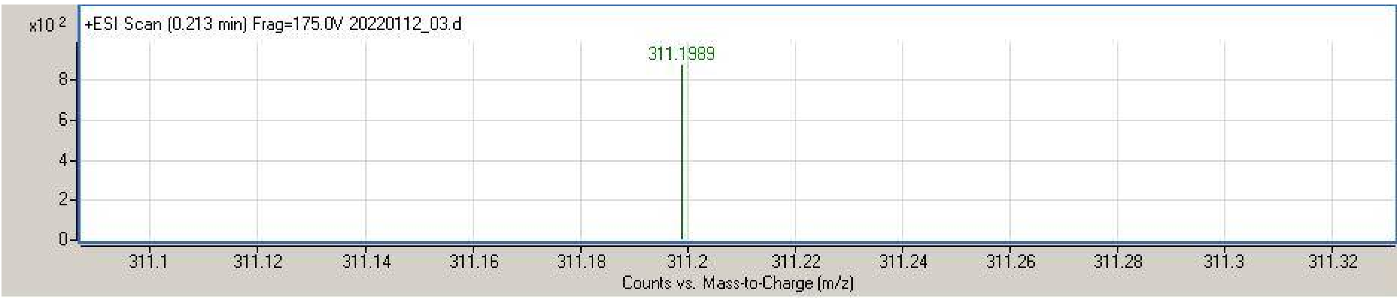

Figure S30. HRMS of compound 7.

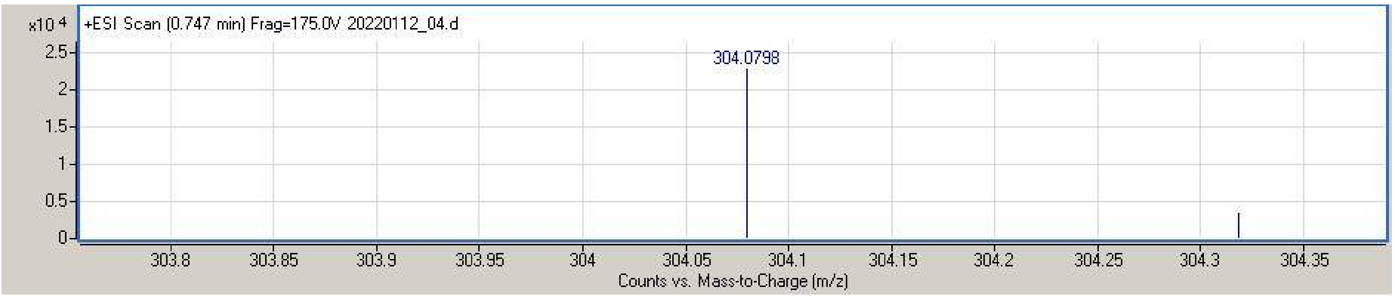

Figure S30. HRMS of compound 8.

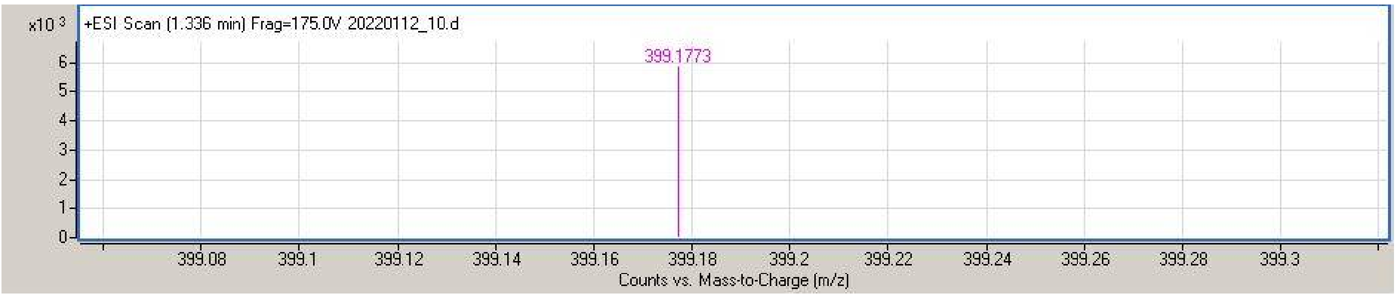

Figure S31. HRMS of compound 12.

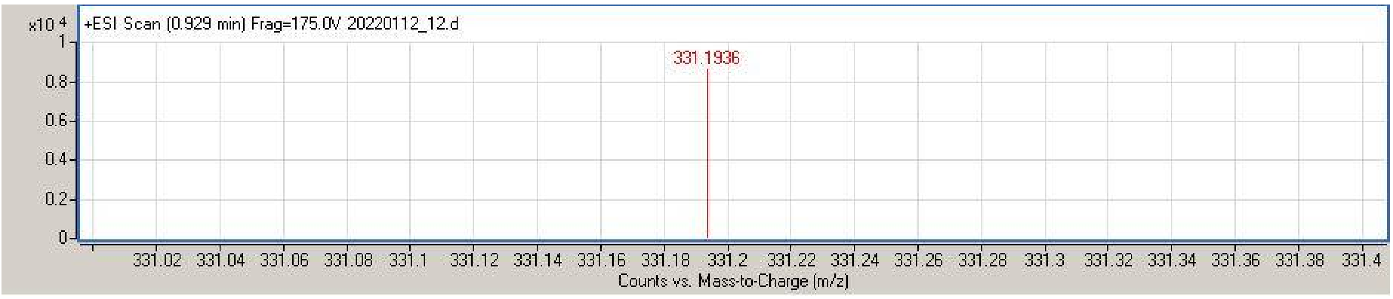

Figure S32. HRMS of compound 14.

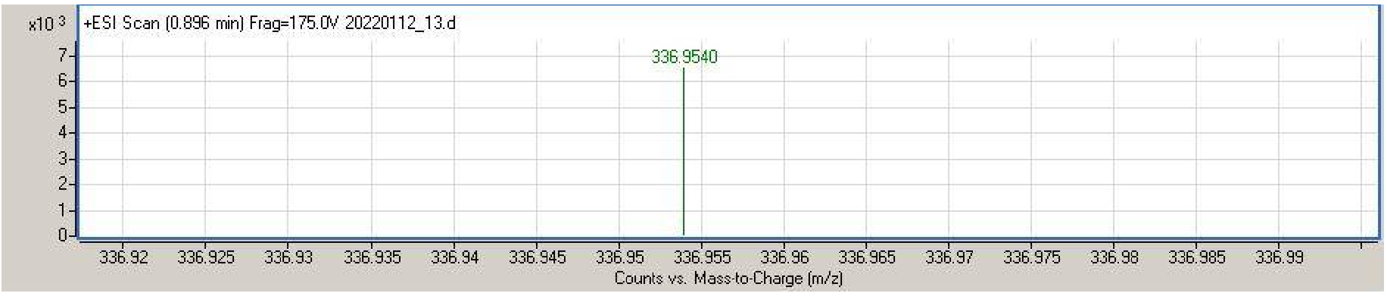

Figure S33. HRMS of compound 15.

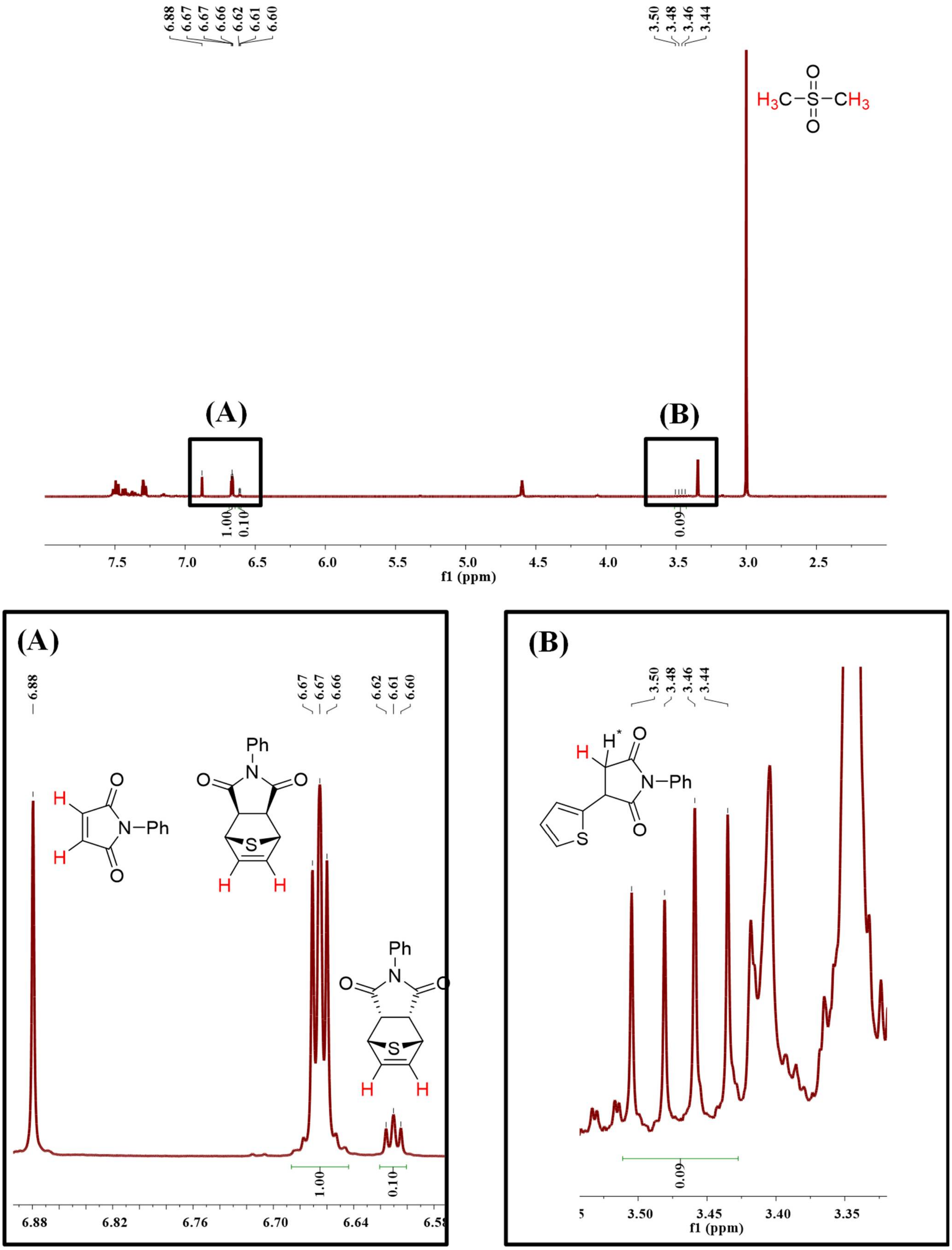

Figure S34. Example of the peak positions for the yield calculations by q-NMR. Left to right: (A) *N*-phenyl maleimide, exo aduct 3, and endo adduct 4; (B) Michael-type adduct 5.

5. NCI´s DTP dose-response report for compound 15.

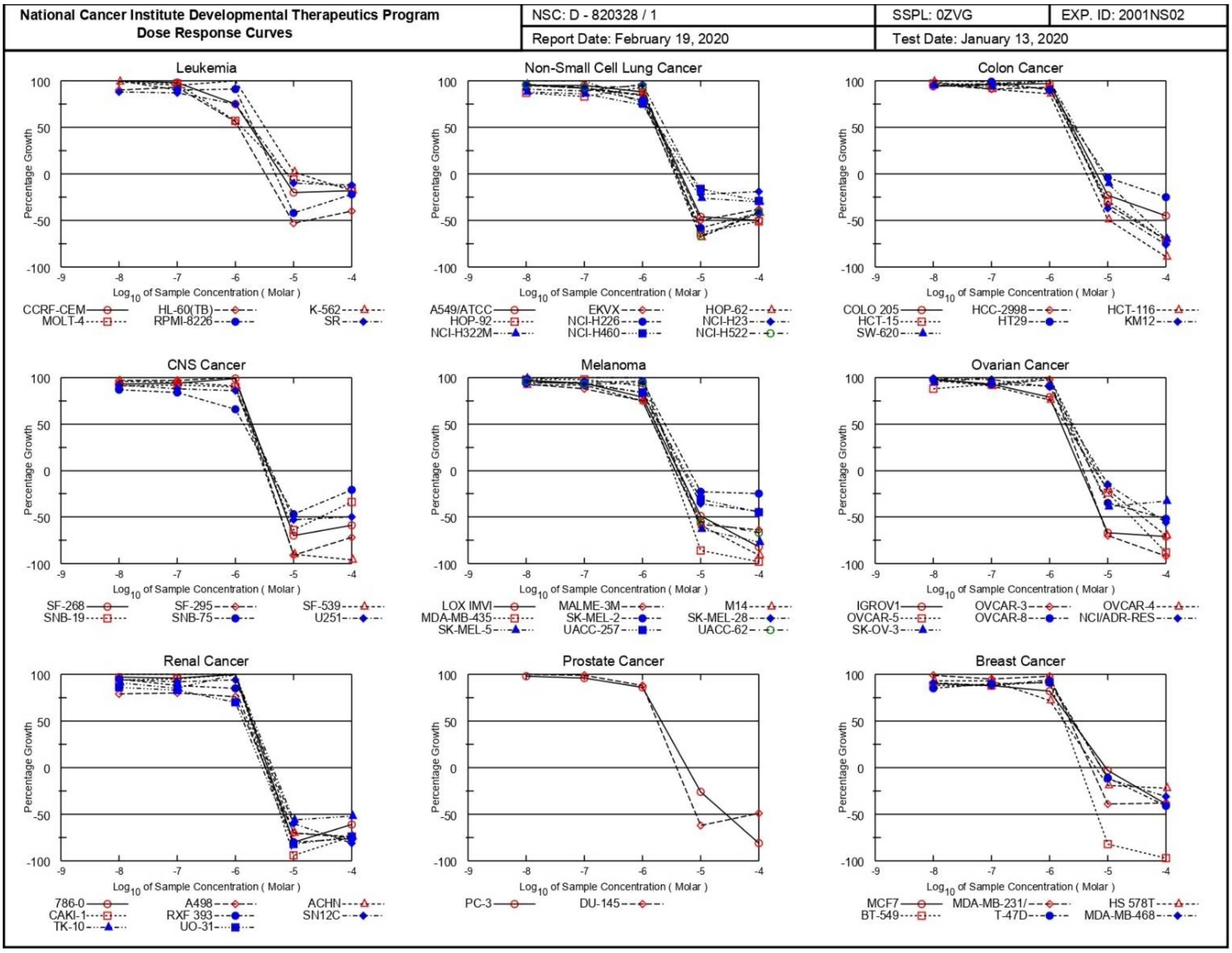

Figure S35. NCI´s DTP dose-response report for compound 15. Page 1 of 4.

National Cancer Institute Developmental Therapeutics Program
In-Vitro Testing Results

NSC : D - 820328 / 1	Experiment ID : 2001NS02	Test Type : 08	Units : Molar
Report Date : February 19, 2020	Test Date : January 13, 2020	QNS :	MC :
COMI : GC-235	Stain Reagent : SRB Dual-Pass Related	SSPL : 0ZVG	

Panel/Cell Line	Time Zero	Ctrl	Mean Optical Densities (Log10 Concentration) -8.0	-7.0	-6.0	-5.0	-4.0	Percent Growth (Log10 Concentration) -8.0	-7.0	-6.0	-5.0	-4.0	GI50	TGI	LC50
**Leukemia**															
CCRF-CEM	0.531	2.065	2.084	2.032	1.684	0.424	0.435	101	98	75	-20	-18	1.84E-6	6.14E-6	> 1.00E-4
HL-60(TB)	0.745	2.860	2.641	2.711	1.926	0.350	0.447	90	93	56	-53	-40	1.13E-6	3.26E-6	.
K-562	0.169	1.591	1.570	1.521	1.599	0.192	0.139	99	95	101	2	-18	3.24E-6	1.20E-5	> 1.00E-4
MOLT-4	0.633	2.780	2.786	2.716	1.866	0.596	0.539	100	97	57	-6	-15	1.31E-6	8.06E-6	> 1.00E-4
RPMI-8226	1.012	2.254	2.251	2.133	2.145	0.587	0.790	100	90	91	-42	-22	2.04E-6	4.84E-6	> 1.00E-4
SR	0.291	1.622	1.462	1.452	1.290	0.263	0.256	88	87	75	-10	-12	1.98E-6	7.70E-6	> 1.00E-4
**Non-Small Cell Lung Cancer**															
A549/ATCC	0.483	2.501	2.397	2.403	2.259	0.263	0.243	95	95	88	-46	-50	1.93E-6	4.56E-6	> 1.00E-4
EKVX	1.070	2.153	2.097	2.061	1.995	0.537	0.666	95	92	85	-50	-38	1.83E-6	4.28E-6	> 1.00E-4
HOP-62	1.222	2.982	2.910	2.847	2.703	0.388	0.712	96	92	84	-68	-42	1.67E-6	3.57E-6	.
HOP-92	1.344	1.983	1.902	1.873	1.938	0.493	0.664	87	83	93	-63	-51	1.88E-6	3.94E-6	8.22E-6
NCI-H226	1.051	1.921	1.887	1.850	1.741	0.444	0.613	96	92	79	-58	-42	1.64E-6	3.79E-6	.
NCI-H23	0.905	2.500	2.364	2.323	2.439	0.706	0.731	91	89	96	-22	-19	2.46E-6	6.51E-6	> 1.00E-4
NCI-H322M	0.998	2.329	2.169	2.142	1.987	0.738	0.698	88	86	74	-26	-30	1.75E-6	5.50E-6	> 1.00E-4
NCI-H460	0.238	2.596	2.658	2.647	2.034	0.201	0.168	103	102	76	-16	-29	1.93E-6	6.77E-6	> 1.00E-4
NCI-H522	1.289	3.131	3.055	3.008	3.016	0.423	0.755	96	93	94	-67	-41	1.87E-6	3.82E-6	.
**Colon Cancer**															
COLO 205	0.661	2.880	2.750	2.785	2.806	0.509	0.363	94	96	97	-23	-45	2.45E-6	6.42E-6	> 1.00E-4
HCC-2998	0.510	2.112	2.052	1.973	1.996	0.340	0.148	96	91	93	-33	-71	2.18E-6	5.44E-6	2.77E-5
HCT-116	0.288	2.425	2.404	2.227	2.125	0.148	0.032	99	91	86	-49	-89	1.85E-6	4.35E-6	1.08E-5
HCT-15	0.299	2.306	2.249	2.220	2.126	0.214	0.088	97	96	91	-29	-71	2.20E-6	5.77E-6	3.24E-5
HT29	0.431	2.642	2.650	2.621	2.422	0.412	0.324	100	99	90	-4	-25	2.65E-6	8.98E-6	> 1.00E-4
KM12	0.387	2.000	1.928	1.952	1.996	0.245	0.091	95	97	100	-37	-76	2.32E-6	5.38E-6	2.16E-5
SW-620	0.414	2.501	2.404	2.380	2.510	0.375	0.125	95	94	100	-10	-70	2.87E-6	8.19E-6	4.69E-5
**CNS Cancer**															
SF-268	1.138	2.871	2.756	2.760	2.849	0.346	0.465	93	94	99	-70	-59	1.95E-6	3.86E-6	7.65E-6
SF-295	0.873	3.180	3.107	3.103	3.184	0.081	0.244	97	97	100	-91	-72	1.83E-6	3.35E-6	6.12E-6
SF-539	0.767	2.406	2.348	2.318	2.265	0.075	0.030	96	95	91	-90	-96	1.69E-6	3.19E-6	6.00E-6
SNB-19	0.550	2.088	1.944	1.960	1.928	0.199	0.363	91	92	90	-64	-34	1.81E-6	3.83E-6	.
SNB-75	0.925	1.816	1.704	1.672	1.516	0.490	0.729	87	84	66	-47	-21	1.39E-6	3.85E-6	> 1.00E-4
U251	0.498	2.498	2.344	2.254	2.208	0.232	0.247	92	88	86	-53	-50	1.80E-6	4.13E-6	9.45E-6
**Melanoma**															
LOX IMVI	0.442	2.979	2.899	2.820	2.437	0.225	0.078	97	94	79	-49	-82	1.67E-6	4.12E-6	1.06E-5
MALME-3M	0.724	1.749	1.676	1.622	1.490	0.302	0.258	93	88	75	-58	-64	1.53E-6	3.65E-6	8.66E-6
M14	0.411	1.202	1.139	1.137	1.007	0.167	0.035	92	92	75	-59	-91	1.54E-6	3.62E-6	8.52E-6
MDA-MB-435	0.640	2.857	2.807	2.806	2.652	0.088	0.015	98	98	91	-86	-98	1.70E-6	3.26E-6	6.24E-6
SK-MEL-2	1.185	2.713	2.619	2.639	2.652	0.915	0.885	94	95	96	-23	-25	2.44E-6	6.43E-6	> 1.00E-4
SK-MEL-28	0.964	2.663	2.582	2.561	2.388	0.619	0.541	95	94	84	-36	-44	1.92E-6	5.02E-6	> 1.00E-4
SK-MEL-5	1.104	3.209	3.190	3.211	3.201	0.406	0.254	99	100	100	-63	-77	2.02E-6	4.09E-6	8.29E-6
UACC-257	1.261	2.759	2.707	2.652	2.515	0.876	0.698	97	93	84	-31	-45	1.97E-6	5.40E-6	> 1.00E-4
UACC-62	0.982	2.925	2.843	2.798	2.813	0.440	0.322	96	93	94	-55	-67	1.98E-6	4.27E-6	9.23E-6
**Ovarian Cancer**															
IGROV1	0.568	2.204	2.166	2.085	1.864	0.188	0.166	98	93	79	-67	-71	1.59E-6	3.49E-6	7.66E-6
OVCAR-3	0.585	1.959	1.939	1.848	1.939	0.178	0.044	99	92	98	-70	-92	1.94E-6	3.86E-6	7.65E-6
OVCAR-4	0.681	1.714	1.681	1.625	1.466	0.540	0.202	97	91	76	-21	-70	1.85E-6	6.10E-6	3.89E-5
OVCAR-5	0.534	1.409	1.302	1.348	1.421	0.404	0.064	88	93	101	-24	-88	2.56E-6	6.40E-6	2.53E-5
OVCAR-8	0.413	2.068	2.031	1.951	1.919	0.268	0.197	98	93	91	-35	-52	2.11E-6	5.26E-6	7.33E-5
NCI/ADR-RES	0.583	2.254	2.232	2.226	2.087	0.495	0.258	99	98	90	-15	-56	2.40E-6	7.18E-6	7.19E-5
SK-OV-3	1.100	2.568	2.498	2.622	2.811	0.667	0.737	95	104	117	-39	-33	2.67E-6	5.59E-6	> 1.00E-4
**Renal Cancer**															
786-0	0.762	2.672	2.618	2.603	2.762	0.149	0.296	97	96	105	-80	-61	1.97E-6	3.68E-6	6.85E-6
A498	1.775	2.523	2.368	2.371	2.345	0.541	0.447	79	80	76	-70	-75	1.51E-6	3.33E-6	7.34E-6
ACHN	0.340	1.507	1.532	1.520	1.570	0.102	0.088	102	101	105	-70	-74	2.07E-6	3.99E-6	7.69E-6
CAKI-1	0.741	2.492	2.389	2.406	2.593	0.046	0.191	94	95	106	-94	-74	1.90E-6	3.39E-6	6.03E-6
RXF 393	1.312	2.112	2.070	2.018	1.993	0.261	0.310	95	88	85	-80	-76	1.63E-6	3.27E-6	6.57E-6
SN12C	0.644	2.647	2.533	2.483	2.520	0.257	0.124	94	92	94	-60	-81	1.92E-6	4.07E-6	8.60E-6
TK-10	0.784	1.804	1.711	1.653	1.842	0.342	0.375	91	85	104	-56	-52	2.16E-6	4.44E-6	9.12E-6
UO-31	0.902	2.051	1.890	1.856	1.711	0.165	0.237	86	83	70	-82	-74	1.36E-6	2.90E-6	6.19E-6
**Prostate Cancer**															
PC-3	0.542	2.078	2.047	2.014	1.870	0.403	0.103	98	96	86	-26	-81	2.11E-6	5.90E-6	2.75E-5
DU-145	0.762	2.543	2.566	2.521	2.331	0.288	0.387	101	99	88	-62	-49	1.79E-6	3.86E-6	.
**Breast Cancer**															
MCF7	0.288	1.875	1.721	1.692	1.589	0.279	0.176	90	88	82	-3	-39	2.37E-6	9.19E-6	> 1.00E-4
MDA-MB-231/ATCC	0.730	1.781	1.766	1.724	1.762	0.446	0.449	99	95	98	-39	-38	2.24E-6	5.20E-6	> 1.00E-4
HS 578T	1.059	2.095	2.024	2.025	1.807	0.860	0.826	93	93	72	-19	-22	1.75E-6	6.21E-6	> 1.00E-4
BT-549	0.955	2.179	2.027	2.034	2.080	0.173	0.033	88	88	92	-82	-97	1.74E-6	3.38E-6	6.56E-6
T-47D	1.039	2.516	2.299	2.372	2.384	0.924	0.617	85	90	91	-11	-41	2.52E-6	7.78E-6	> 1.00E-4
MDA-MB-468	0.864	2.025	1.916	1.883	1.957	0.761	0.594	91	88	94	-12	-31	2.61E-6	7.71E-6	> 1.00E-4

Figure S36. NCI´s DTP dose-response report for compound 15. Page 2 of 4.

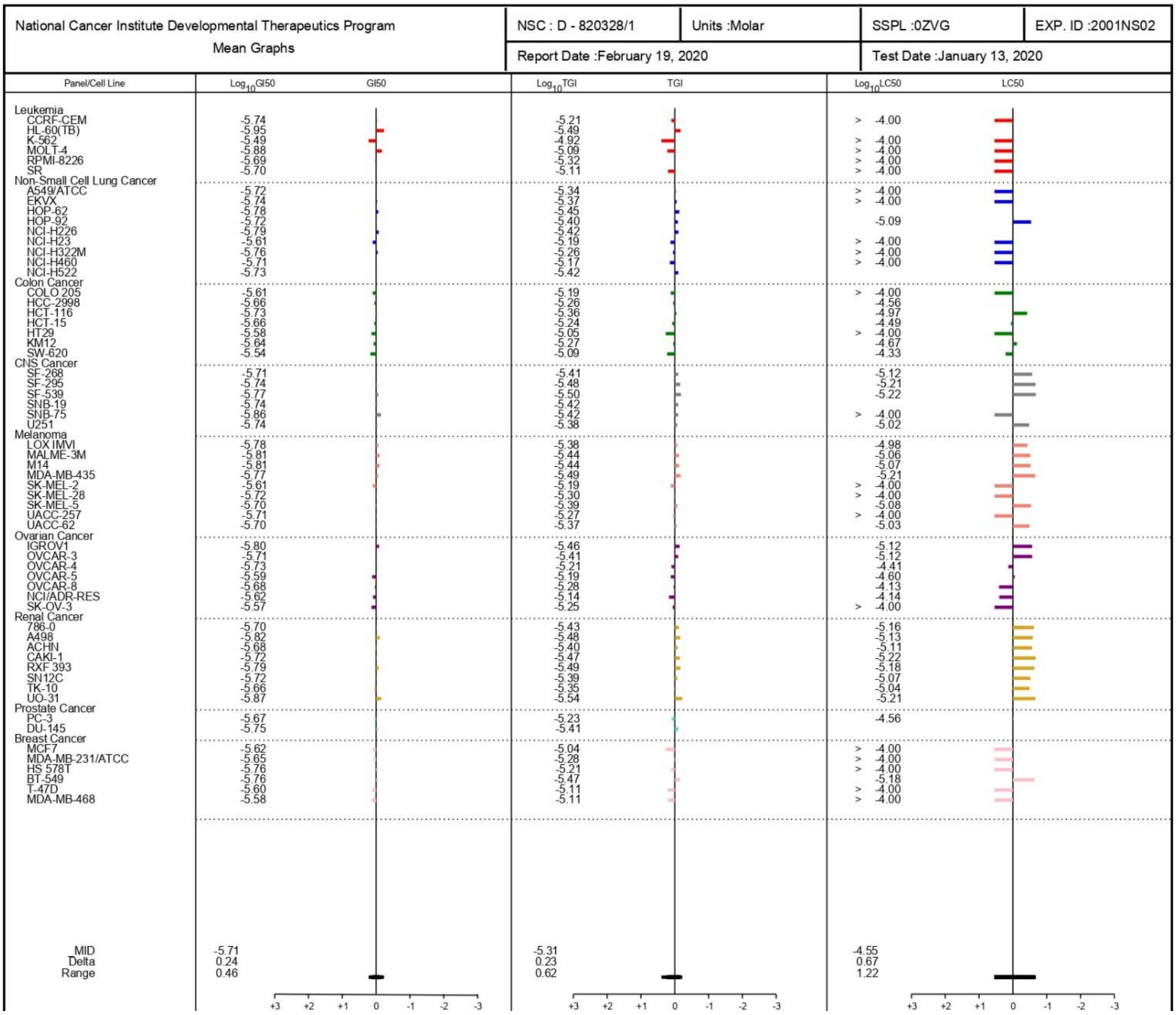

Figure S37. NCI´s DTP dose-response report for compound 15. Page 3 of 4.

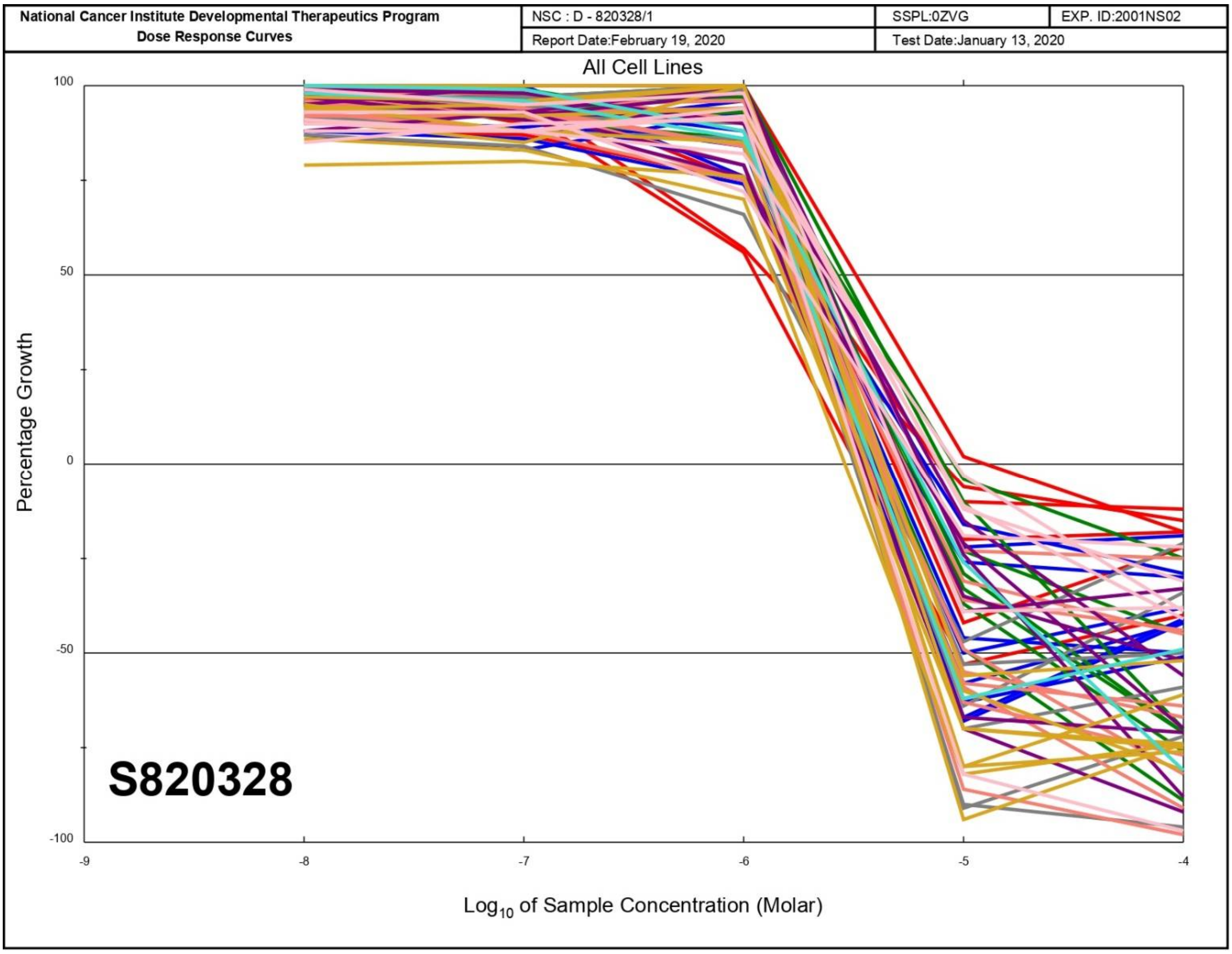

Figure S38. NCI´s DTP dose-response report for compound 15. Page 4 of 4.
